# Unmasking Novel Loci for Internal Phosphorus Utilization Efficiency in Rice Germplasm through Genome-Wide Association Analysis

**DOI:** 10.1371/journal.pone.0124215

**Published:** 2015-04-29

**Authors:** Matthias Wissuwa, Katsuhiko Kondo, Takuya Fukuda, Asako Mori, Michael T. Rose, Juan Pariasca-Tanaka, Tobias Kretzschmar, Stephan M. Haefele, Terry J. Rose

**Affiliations:** 1 Crop, Livestock and Environment Division, Japan International Research Centre for Agricultural Science, Tsukuba, Ibaraki, Japan; 2 School of Chemistry, Monash University, Clayton, Victoria, Australia; 3 International Rice Research Institute (IRRI), Los Banos, Philippines; 4 Australian Centre for Plant Functional Genomics (ACPFG), Glen Osmond, South Australia, Australia; 5 Centre for Plant Sciences, Southern Cross University, Lismore, New South Wales, Australia; Institute of Botany, Chinese Academy of Sciences, CHINA

## Abstract

Depletion of non-renewable rock phosphate reserves and phosphorus (P) fertilizer price increases has renewed interest in breeding P-efficient varieties. Internal P utilization efficiency (PUE) is of prime interest because there has been no progress to date in breeding for high PUE. We characterized the genotypic variation for PUE present within the rice gene pool by using a hydroponic system that assured equal plant P uptake, followed by mapping of loci controlling PUE via Genome-Wide Association Studies (GWAS). Loci associated with PUE were mapped on chromosomes 1, 4, 11 and 12. The highest PUE was associated with a minor *indica*-specific haplotype on chromosome 1 and a rare *aus*-specific haplotype on chromosome 11. Comparative variant and expression analysis for genes contained within the chromosome 1 haplotype identified high priority candidate genes. Differences in coding regions and expression patterns between genotypes of contrasting haplotypes, suggested functional alterations for two predicted nucleic acid-interacting proteins that are likely causative for the observed differences in PUE. The loci reported here are the first identified for PUE in any crop that is not confounded by differential P uptake among genotypes. Importantly, modern rice varieties lacked haplotypes associated with superior PUE, and would thus benefit from targeted introgressions of these loci from traditional donors to improve plant growth in phosphorus-limited cropping systems.

## Introduction

Global food security depends on the availability and affordability of inputs such as phosphorus (P) fertilizers. Given that rock phosphate reserves used to produce P fertilizers are non-renewable, the price of P fertilizer will likely increase over the coming decades as the readily accessible, high-grade rock phosphate reserves are depleted and the price of oil rises [1]. A large proportion of the world’s resource-poor farmers in developing countries cannot afford P fertilizers at present, and to prevent them from falling behind even further, there has been a global push to reduce agronomic P fertilizer requirements by breeding cultivars that can utilize P more efficiently—herein referred to as P-efficient crops [2]. A further incentive to breed P-efficient crops is the fact that the inefficient P fertilizer use is a major cause of environmental pollution through the eutrophication of waterways [3].

Phosphorus efficiency has typically been described by two separate components: P acquisition efficiency (PAE) and internal P utilization efficiency (PUE) [4]. PAE relates to P uptake from the soil and may be affected at the genotype level by differences in root size, root architecture or rhizosphere interactions that enhance P bioavailability (for details the reader is referred to a recent review, [5]). PUE on the other hand measures how efficiently the P taken up is utilized to accumulate either grain yield or vegetative biomass. The size of different plant P pools and the efficacy with which P is allocated to and remobilized from different pools and tissues over time largely determines PUE. The reader is refereed to recent reviews for detailed discussions of the subject [6][7].

While both aspects of P efficiency have been the subject of considerable research, most advances in understanding and breeding have involved PAE [8]. One recent breakthrough has been the identification of a protein kinase gene ‘*Phosphorus*-*Starvation Tolerance 1*’ (*OsPSTOL1*) in rice that confers enhanced PAE through sustained root growth in low-P soils [9]. Markers diagnostic for *OsPSTOL1* are currently employed in marker-assisted breeding of P efficient rice [10].

In contrast, PUE has received less attention and markers are not available for breeding crops with enhanced PUE [7]. Two approaches have been employed to investigate PUE: The first has been to investigate physiological and transcriptional changes in P-starved plants. Numerous traits termed ‘adaptations to P starvation’ have been identified, including avoidance of glycolytic pathways that rely heavily on P, increased scavenging/recycling of inorganic P from organic P compounds (e.g. by PAPases), and replacement of phospholipids by sulfolipids [11].While these responses represent typical changes occurring under P deficiency, there is no indication that genotypic variation for such traits exists that could be exploited in plant improvement; indeed, in rice the typical transcriptional changes observed under P deficiency are possibly more pronounced in P-inefficient than highly P-efficient genotypes [12].

The second approach has been to screen a range of genotypes for differences in PUE by assessing a suite of P-efficiency criteria simultaneously (PAE, PUE, grain yield per unit P uptake etc.) on the premise that doing so provides a more comprehensive insight into mechanisms underlying crop P efficiency [13,14]. The danger of such an approach lays in the complexities and interactions among P efficiency criteria; measuring them concurrently may mask the true effect of any individual PUE trait [15]. Any P efficiency criterion involving grain yield may be dominated by effects of harvest index if genotypes differ markedly in that trait [16]. In screening experiments conducted under P-deficient conditions in the field or in media that expose genotypic differences in P acquisition, any genotype with better P uptake is likely to suffer less P deficiency stress, which in turn will affect its physiological response [7].

The *Pup1* locus illustrates this point: Being the most influential QTL mapped for tolerance to P deficiency to date, it was subsequently shown to enhance P uptake via enhanced root growth [9]. However, it had also been mapped as a major QTL for PUE [17], but while donor ‘Kasalath’ enhanced P uptake it appeared to reduce PUE. The authors [17] concluded that the PUE QTL should be treated as a pseudo-QTL; a consequence of poor P uptake of lines lacking the Kasalath *Pup1* locus that subjects them to higher levels of P starvation, which in turn gives the impression of having produced more biomass per unit P taken up (at very low total biomass and P uptake). Similar interactions between P uptake and PUE are observed in most screening studies, and several ‘pseudo-QTLs’ for PUE have been subsequently mapped (reviewed by [7]). Simultaneous screening of PUE and PAE may thus explain why little progress in breeding crops with high PUE has been achieved to date [15].

To avoid confounding effects of P uptake on PUE, Rose *et al*. [15] screened genotypes in hydroponics at equal P uptake, and showed that genotypic rankings in PUE are completely different to those obtained in trials allowing P uptake to differ between genotypes. Equal P uptake was achieved by growing genotypes individually in containers at a fixed level of P supply that was low (deficient) enough to be rapidly taken up by each genotype [15]. Having comparable P content, differences in plant biomass were thus due to efficient redistribution and utilization of P taken up.

The objective of the present study was to employ the concept of screening at equal plant P content to map loci controlling PUE in rice. Rather than relying on a bi-parental mapping population and the limited genetic variation contained within, a rice association panel of 292 genotypes of diverse plant type and origin that represent all five subpopulations of *Oryza sativa* [18] was used. Because the plant material varied considerably for yield potential, harvest index and maturity class, which would confound PUE estimates based on grain yield, the study focused solely on PUE at the vegetative growth stage.

## Results

### Plant biomass, P utilization efficiency and P uptake

In Exp1 plants received a single dose of 800 μg P at 10 DAS (S1 Fig), nearly all of which was taken up within 5 days (S1 Table). Beyond 14 DAS plants showed signs of P deficiency: visibly reduced growth and fewer leaves than high-P plants (Table 1). The trait most affected by P deficiency was the number of green leaves, while dry leaf number and plant height were least affected. Exp2 differed in that P was supplied in small doses throughout the growth period (S1 Fig) to a total of 1550 μg P, which enabled plants to accumulate more biomass compared to Exp1 (Table 1). High-P control plants were also bigger in Exp2 compared to Exp1 which was due to having entered the exponential growth phase during mid-tillering. One week earlier, differences between both treatments were smaller and comparable to Exp1 (data not shown).

**Table 1:** Average and relative plant performance in experiment 1 and experiment 2.

	Leaf number			Height	Tillers	Biomass
	total	green	dry	cm		g plant^-1^
**EXP 1**						
low P	8.4	3.6	4.8	51.8	1.3	1.04
high P	12.2	7.1	5.1	65.2	1.8	1.54
relative (%)	68.9	50.7	94.1	79.4	72.2	67.5
**EXP 2**						
low P	8.1	4.9	3.2	75.7	1.1	1.28
high P	20.6	18.3	2.3	99.1	4.0	3.74
relative (%)	39.3	26.8	139.1	76.4	27.5	34.2

Because nearly all P was taken up within 5 days (S2 Fig), all genotypes had comparable P content and PUE was estimated as total biomass per amount of P acquired (∑ P supplied and seed P). PUE (inverse of tissue-P concentration) measured in a subset of genotypes was closely related to estimated PUE (R^2^ = 0.86 in Exp1 and 0.74 in Exp2; S3 Fig), confirming that the PUE estimates were valid. This eliminated the need for labor-intensive tissue P concentration measurements in hundreds of samples.

**Fig 1:**
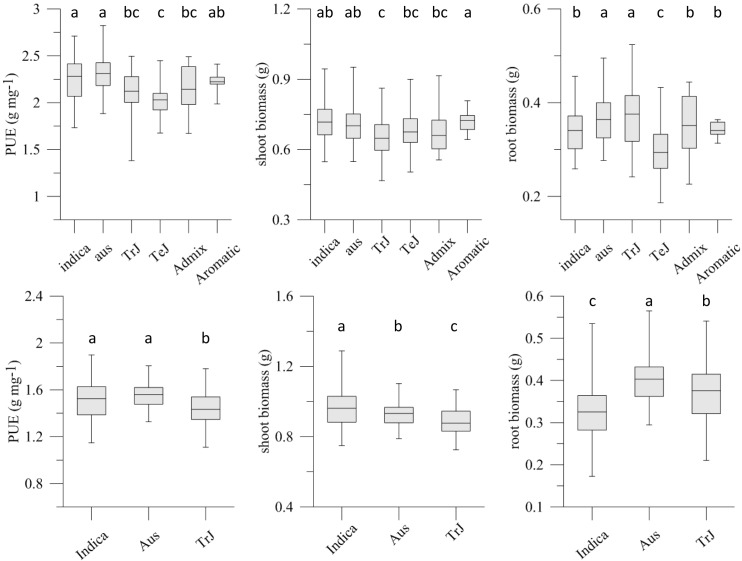
Variation within subpopulations for PUE and shoot and root biomass in experiment 1 and experiment 2. Top panels show data from experiment 1: indica, n = 66; aus, n = 46; TrJ, n = 78; TeJ, n = 77; Admix, n = 13; Aromatic, n = 11; bottom panels show data from experiment 2: indica, n = 71; aus, n = 58; TrJ, n = 69. Less variable subpopulations and those with few members were omitted in experiment 2. Letters above error bars indicate significant differences between subpopulation means (Tukey’s HSD, P < 0.05). TrJ and TeJ refer to tropical and temperate *japonicas*, respectively.

PUE was higher in Exp1 than Exp2 (Fig 1), and average PUE in Exp1 was highest in the *indica* and *aus* subpopulations and lowest in temperate *japonicas* (TeJ). Since overall variability was also lowest in TeJ they were excluded from Exp2 as was the small group of aromatic rices. ‘Admix’ accessions were included if they could be classified as predominantly *indica*, *aus*, or tropical *japonica* (TrJ). While overall PUE was higher in Exp1, the comparison of within and between subpopulations agreed well between experiments (Fig 1). On average, TrJ accessions had lower PUE than *aus* and *indica* due to lower shoot biomass. For root biomass TrJs were similar to the *aus* subpopulation and superior to *indicas*. The correlation between PUE values in both experiments was r = 0.49 (S2 Table).

In Exp1 accession Coarse achieved the highest PUE (2.82 g biomass mg^-1^ P), followed by Yodanya, Seratoes Hari (all *indica*) and Santhi Sufaid (*aus*) (see S2 Table for genotypic means). In Exp2 the top performers were accessions Guan-Yin-Tsan, Tsipala421 (*indica*) and Santhi Sufaid (*aus*). In Exp1 lowest PUE values of 1.38 and 1.53 g biomass mg^-1^ P were detected in accessions Chahora144 and IRAT13 (TrJ), followed by several TeJ. Among *indicas*, Teqing and IR8 had low PUE (1.73 and 1.79, respectively) and they were also the lowest *indicas* in Exp2, whereas Saku and Cocodrie were the least efficient TrJ. Overall, modern semi-dwarf varieties had below-average PUE while most highly efficient accessions were traditional types.

Estimated PUE was tightly correlated to total dry weight (TDWt) and less so with shoot or root biomass in both experiments (Table 2). Stem biomass had a lower correlation than leaves and roots in Exp1. In Exp2, stem and leaf material wasn’t separated and combined shoot weight had a higher correlation (r = 0.86) with PUE (and TDWt) than root weight (r = 0.72). Plant height was positively correlated with PUE and TDWt but the effect of height was smaller than for shoot or root biomass (Table 2). Correlations between seed P content and PUE or TDWt were non-significant or low (Table 2), indicating that genotypic differences in seed P content didn’t affect results. P redistribution from old to new leaves was estimated as the proportion of leaves or leaf biomass made up of dry leaves (DL No (%) and DL Wt (%)), but neither estimate contributed substantially to variation for PUE. The root/shoot ratio had either no significant (Exp1) relationship or only a minor relationship with PUE (Exp2, r = 0.33).

**Table 2:** Correlation coefficients between main traits in Exp1 and Exp2.

	PUE	Total weight	Leaf weight	Stem weight	Root weight	Seed P	Dry leaf number	Dry leaf weight	Height	*+P*
**Exp1** ^**a**^										
TDWt	0.94									*0*.*45*
LeafWt	0.67	0.68								*0*.*63*
StemWt	0.47	0.52	-0.05							*0*.*58*
RootWt	0.60	0.61	0.52	-0.23						*0*.*70*
Seed-P	-0.26	0.09	-0.03	0.12	-0.07					*-*
DL^c^ No (%)	0.12	0.16	0.21	-0.09	0.04	0.11				*0*.*21*
DL^c^ Wt (%)	0.12	0.21	0.25	0.04	-0.07	0.26	0.73			*0*.*27*
Height	0.49	0.53	0.54	0.22	0.16	0.06	0.32	0.29		*0*.*67*
Root:Shoot ratio	0.09	0.05	0.15	-0.65	0.82	-0.14	-0.05	-0.22	-0.16	*0*.*78*
**Exp2** ^**b**^										
TDWt	0.98									*0*.*63*
ShootWt	0.86	0.86								*0*.*70*
RootWt	0.72	0.75	0.31							*0*.*52*
Seed-P	0.08	0.26	0.16		0.29					*-*
DL^c^ No (%)	0.36	0.37	0.29		0.31	0.10				*0*.*01*
TL^c^ No	-0.09	-0.12	-0.03		0.20	-0.20	-0.27			*0*.*45*
Height	0.60	0.58	0.53		0.39	0.02	0.35	-0.11		*0*.*82*
Root:Shoot ratio	0.33	0.37	-0.15		0.89	0.23	0.19	-0.20	0.15	*0*.*61*

Data in the last column shows correlations between same traits in both P treatments.

^a^Statistical parameters: n = 264, P < 0.05 at r = 0.12

^b^Statistical parameters: n = 198, P < 0.05 at r = 0.14

^c^Dry leaf (DL); total leaf (TL). Number (No) and weight (Wt) of DL was calculated as a percentage of TL.

### Genome wide association mapping of *PUE* loci

QQ plots suggested the GLM option overestimated significance (S4 Fig) leading to a potentially high number of false positives in both experiments, whereas MLM may have underestimated effects in Exp2, increasing the probability of false negatives [18]. Our phenotyping shared one common trait (plant height) with the study of Zhao *et al*. [18] that allowed direct comparisons. We identified two major plant height loci on chromosome 1 (33.1 and 38.1–38.5 Mb; S3 Table), corresponding to the exact location of most significant peaks in [18] and to the location of the main dwarfing gene (*sd1*) in rice (38.3 Mb). Further, we detected common minor loci. That we identified major and minor loci across experiments and P treatments with an almost identical level of significance (p < 1.0E-8 for *sd1*) confirms the robustness of statistical procedures used.

**Fig 2:**
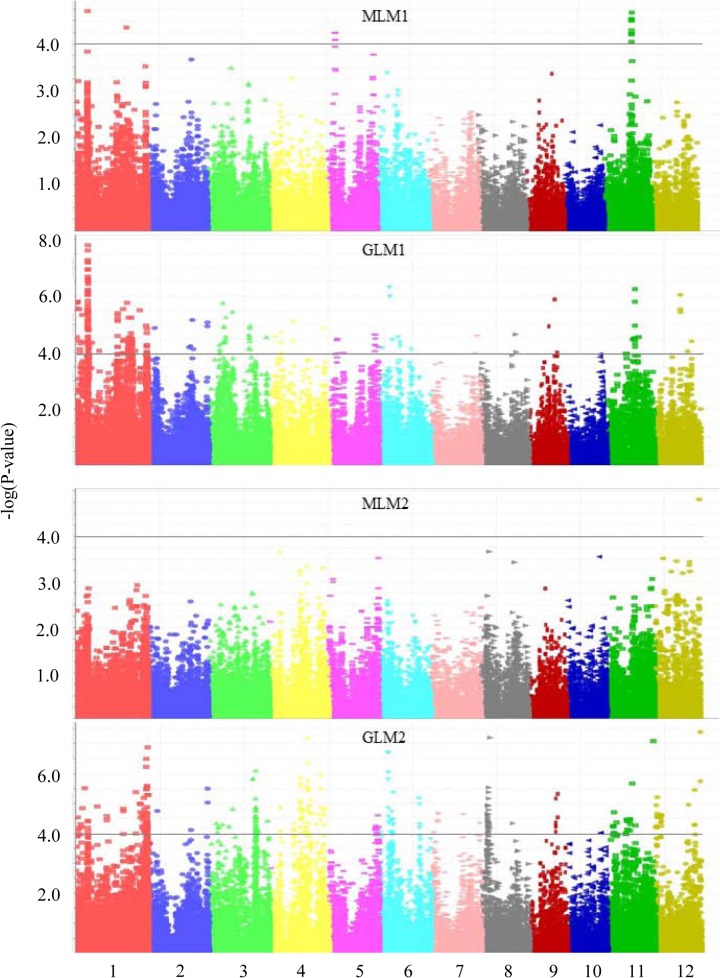
Manhattan plots for PUE (total biomass/total P) based on fixed (GLM) and mixed model (MLM) calculations in experiments 1 and 2. Colors indicate different chromosomes while the horizontal lines indicate a common significance level of P = 0.0001 [-log(P) = 4.0].

In Exp1 main loci for PUE were detected on chromosomes 1, 5 and 11 with good agreement between MLM and GLM (Table 3; Fig 2). In Exp2 only one locus exceeding P < 1.0E-04 was detected using MLM (chromosome 12) while GLM identified peaks on all chromosomes. Table 3 summarizes main loci identified across experiments based on an analysis across all subpopulations as this typically produced most significant peaks. The exception is the locus on chromosome 4 where an *indica*-only analysis enhanced significance by two orders of magnitude. Two peaks on chromosome 1 (7.3 Mb) and chromosome 11 (16.7 Mb) were discerned across experiments. To determine whether variation for root or shoot biomass contributed over-proportionally to PUE peak significance, further analyses using shoot, root or total biomass as traits were conducted. Results indicate that peaks for PUE on chromosomes 4 (17.7 Mb) and 12 (26.6 Mb) were associated with variation for shoot biomass while peaks on chromosomes 1 (30.5 Mb) and 5 (4.3 Mb) were associated with variation for root biomass (Table 3). The remaining loci were most significant for total plant biomass. Given that overall PUE was less tightly correlated with root biomass we consider PUE peaks attributed to variation in total biomass and shoot biomass as being the main loci of interest. Of *PUE* loci detected in the low-P treatments in Exp1, only the peak on chromosome 11 was also significant for non-stress biomass. This peak also appeared in the high-P treatment in Exp2 as did three other loci (Table 3).

**Table 3:** Loci detected as being associated with PUE in experiments 1 and 2.

		Experiment 1				Experiment 2				
Chr.	Interval ^a^ (Mb)	MLM	GLM	MLM (+P)	sub population	MLM	GLM	MLM (+P)	sub population	biomass trait ^b^
1	7.22–7.37	2.0E-05	1.6E-08	nd	TrJ/*indica*	1.4E-03	1.8E-05	4.4E-03	*indica*	total
1	30.5	4.4E-05	1.7E-06	nd	TrJ	nd	nd	nd	*-*	root
4	17.70–17.85	nd	nd	nd	-	2.0E-06 ^c^	1.4E-06	nd	*indica*	shoot
5	4.33–4.56	5.7E-05	3.4E-05	nd	TrJ	nd	nd	nd	*-*	root
11	16.63–16.75	2.1E-05	5.7E-07	7.9E-04	*aus*	2.2E-03	5.5E-05	3.0E-03	*aus*	total
12	26.61–26.66	nd	nd	nd	-	1.5E-05	9.7E-09	1.9E-05	*indica*	shoot

^a^Interval size estimate base on linkage blocks present at respective locus (supplementary S5 Fig)

^b^At each PUE peak we examined whether variation for root or shoot biomass contributed over-proportionally to peak significance.

^c^Result of an analysis including only accessions of the indica subspecies (n = 65). The significance level decreased to 6.2E-04 for the analysis across subpopulations.

TrJ = tropical *japonica*.

‘nd’ signifies ‘not detected’

Significance levels of main peaks were highest when analyzed across all subpopulations in Exp1; however, variation within TrJ contributed largely to peaks on chromosomes 1 and 5, while variation within *aus* contributed to the peak on chromosome 11 (Table 3). In Exp2, subpopulation effects were highly significant. Variation within *indica* was particularly influential on chromosome 4 (17.8 Mb) and on chromosomes 1 and 12 (Table 3). As in Exp1 the *aus* subpopulation contributed to the QTL on chromosome 11. No peaks in Exp1 could be attributed to variation within TeJ, probably because variability was lower compared to other main subpopulations (Fig 1).

### Haplotype analysis of main loci

**Fig 3:**
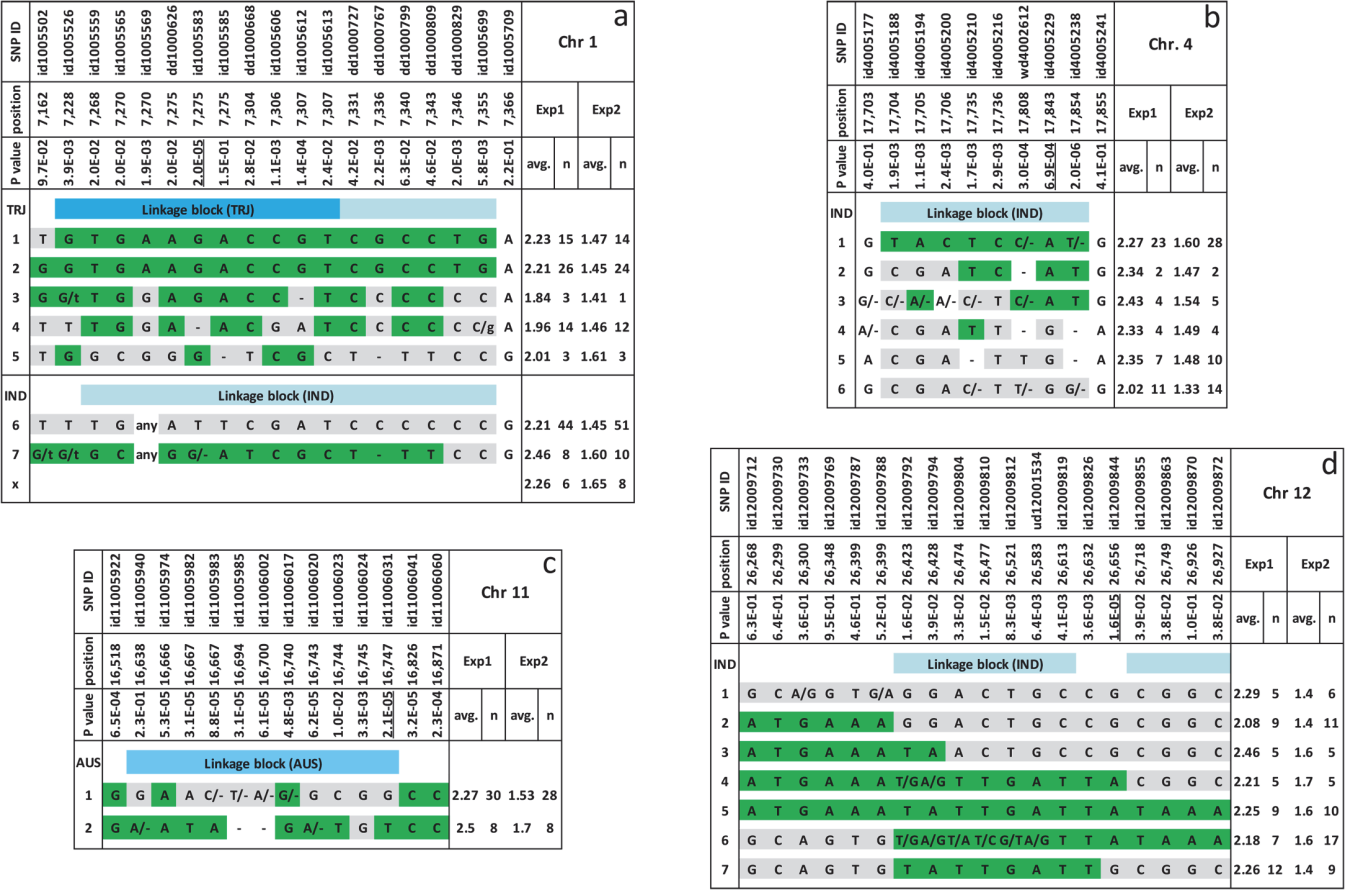
Haplotype analysis for main loci associated with PUE on chromosomes 1 (A), 4 (B), 11 (C), and 12 (D). Within haplotypes most significant SNPs highlighted in bold print and average PUE values of accessions belonging to respective haplotype are given. SNP alleles improving PUE are highlighted in green while negative alleles are highlighted grey. Linkage blocks for the respective subpopulation are indicated in blue above the haplotypes (see S5 Fig for details on LD analysis).

Genotypic variation patterns around most significant peaks were further investigated using sub-population-specific LD analysis to estimate boundaries of a particular locus. The peak at 7.27 Mb on chromosome 1 was delimited by a strong linkage block between 7.22 and 7.31 Mb for TrJ and by a weaker block from 7.24 to 7.36 Mb for *indicas* (Fig 3A; S5 Fig). Polymorphic patterns within TrJ accessions distinguished two major haplotypes (1 and 2) that were associated with high PUE (Fig 3A) and three minor haplotypes (3–5) associated with below-average PUE (Exp1). For *indicas* two haplotypes were identified. Relative to the major haplotype (HT1-6), the minor haplotype 7 (HT1-7) increased PUE by 11% and 10% in experiments 1 and 2, respectively. Several *indica* accessions did not belong to a specific haplotype and this group ‘x’ showed highest PUE in Exp2.

The peak on chromosome 4 (17.85 Mb) corresponded to an *indica*-specific linkage block between 17.70–17.85 Mb (S5 Fig) with six distinct haplotypes (Fig 3B). Haplotype 1 was most common and associated with high PUE whereas haplotype 6 showed lowest PUE. The *aus*-specific locus on chromosome 11 was located within a small linkage block between 16.63–16.75 Mb (Fig 3C; S5 Fig). Two distinct haplotypes were identified and the minor was associated with high PUE. Another *indica*-specific locus on chromosome 12 had a narrow peak at 26.656 Mb (Exp 2) not located within a linkage block but flanked by two blocks (S5 Fig). Seven haplotypes existed and minor types 3 and 4 associated with superior PUE (Fig 3D).

**Fig 4:**
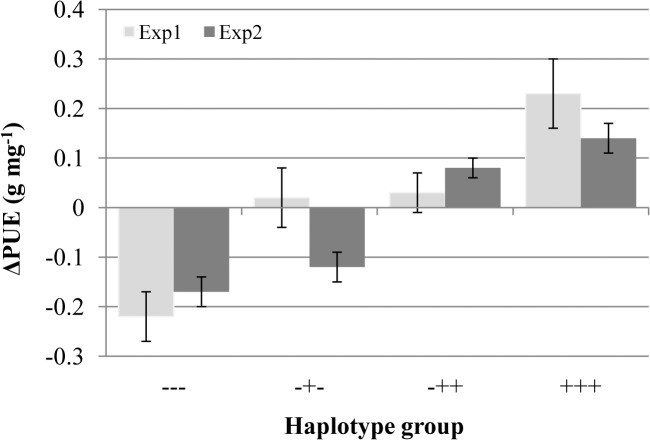
Deviation from the mean PUE for haplotype groups within the *indica* subpopulation. Labels on the x-axis (---,-+-, etc.) refer to having the negative or positive haplotype at each of the QTLs on chromosomes 1 (7.2Mb), 4 (17.8Mb) and 12 (26.6Mb), in that order (see Fig 3). Accessions in the ‘+++’ group include ‘Mudgo’, ‘Short Grain’ and ‘Yodanya’ while the ‘---‘ group are mostly older ‘modern’ varieties (IR8, Taichung Native, Teqing, Zhenshan2). The successful second generation green revolution varieties ‘IR36’ and ‘IR64’ belong to the ‘-+-’ group.

Three significant *PUE* loci were identified in the *indica* subpopulation (Fig 3), allowing for combined haplotype analysis (Fig 4): Having the unfavorable haplotype at three loci (---) for chromosomes 1 (7.2 Mb), 4 (17.8 Mb) and 12 (26.6 Mb) resulted in below-average PUE. Having at least one favorable haplotype (-+-) improved PUE to the overall average level while possessing three favorable haplotypes (+++) resulted in the best performance. The locus on chromosome 12 was only detected in Exp2 (Table 3) and hence, having a second positive haplotype on chromosome 12 (-++) improved performance in Exp2 but not in Exp1. Overall data suggest these three loci act at least partly in an additive manner.

### Candidate genes

**Fig 5:**
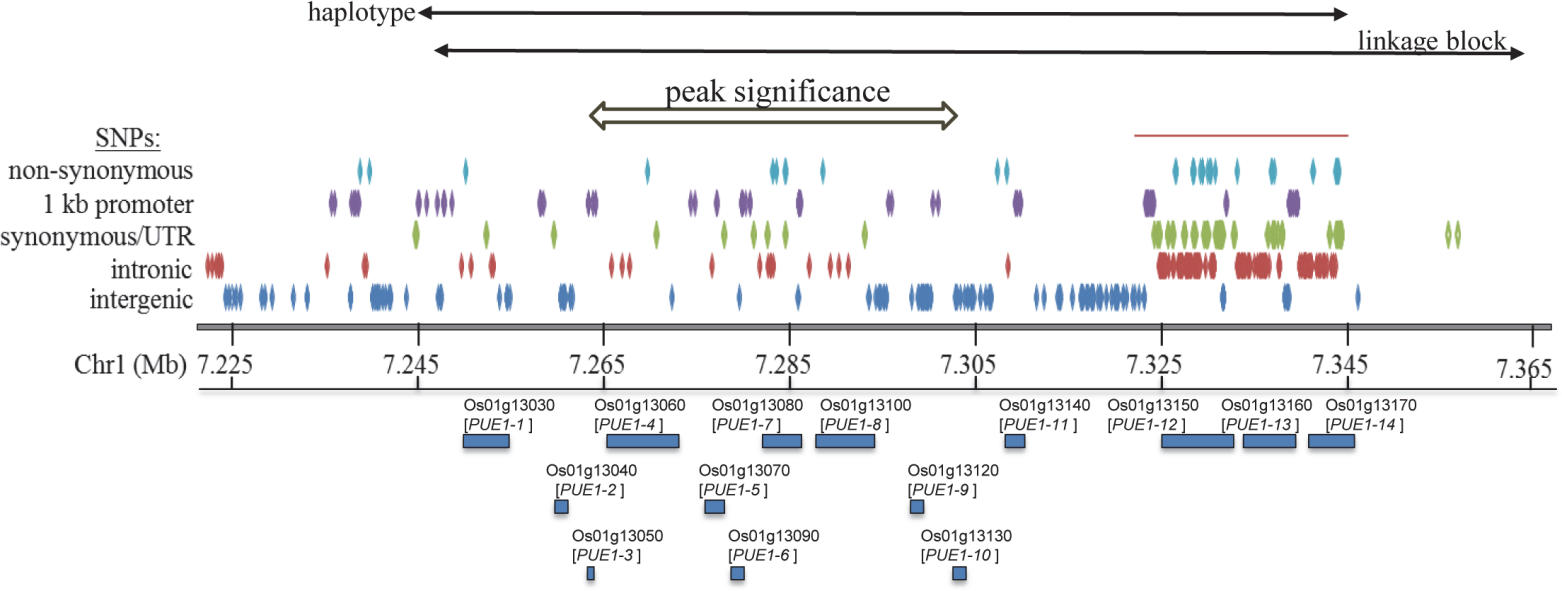
In silico investigation of SNP variation and predicted gene models (MSU6.1) across the indica specific PUE locus on chromosome1, delimited by haplotype and linkage block to 7.247–7.343 Mb. The location of SNP locations with most significant association to PUE is indicated. SNP patterns shown are based on 1479 accessions represented in the RiceVarMap database and indicate specific SNPs detected in accessions belonging to the rare high-PUE haplotype (HT1-7) group (haplotype frequence of 7.3%). A total of 550 SNPs are displayed, of which 332 SNPs cluster towards the downstream limit (red bar on top).

LD and haplotype analyses defined chromosomal regions likely containing causal variation on chromosomes 1, 4, 11 and 12 (Fig 3 and S5 Fig). The 100 kb region on chromosome 1 contained 14 predicted gene loci, some of which had been previously identified in microarray studies [12,19] as being regulated by P deficiency (Fig 5; S5 Table). QTL regions on chromosomes 4 and 11 contained 21 and 24 predicted gene loci, respectively (S6 and S7 Tables). The QTL on chromosome 12 was not defined by a clear linkage block; thus, all gene models in a 500 kb flanking region are listed (S8 Table). Taking predicted function and reported P-responsiveness into account, a priority list of candidate genes that merit further investigation was drafted (Table 4).

**Table 4:** PUE candidate genes detected based on converging evidence from functional annotation, P-responsiveness based on gene expression profiling and SNP variation in the promoter and coding regions.

Chr.	MSU_LOC:	MSU_5'	MSU_3'	MSU_Annotation	Pariasca-Tanaka et al. (2010)	Zheng et al. (2009)	Number of NS-SNPs^b^	Number of P-SNPs^c^
1	LOC_Os01g13030	7248761	7253629	OsIAA3, Auxin-responsive Aux/IAA gene family member, expressed	0.79** S^a^	0.74* S	1	4
	LOC_Os01g13060	7264228	7272237	CK1_CaseinKinase_1.1—CK1, expressed	0.53* S	NS	1	5
	LOC_Os01g13080	7279250	7277720	60S acidic ribosomal protein, putative, expressed	2.16*** S	1.65* R	0	2
	LOC_Os01g13090	7280734	7284992	nucleic acid binding protein, putative, expressed	NS	NS	4	8
	LOC_Os01g13150	7324145	7331536	metallo-beta-lactamase family protein, putative, expressed	na	1.94* R; 2.99* S	14	16
	LOC_Os01g13160	7332691	7338244	expressed protein	0.81** R	NS	4	2
	LOC_Os01g13170	7339717	7344465	ubiquitin-conjugating enzyme E2, putative, expressed	NS	NS	6	15
4	LOC_Os04g30110	17790725	17789889	wall-associated receptor kinase 3 precursor, putative, expressed	na	1.24*** S	ND	ND
11	LOC_Os11g29470	16634045	16631968	expressed protein	na	na	4	4
	LOC_Os11g29490	16642596	16649451	plasma membrane ATPase, putative, expressed	NS	NS	2 (stop codon)	2
12	LOC_Os12g42970	26659915	26662912	GATA zinc finger domain containing protein, expressed	NS	1.86* R	ND	ND
12	LOC_Os12g43110	26734618	26735291	OsSAUR58, Auxin-responsive SAUR gene family member, expressed	NS	1.8* S; 2.6** R	ND	ND

^a^Values above 1 indicate up-regulation of the gene under P deficiency.

Asterisks indicate statistical significance, where * P<0.05, ** P<0.01, *** P<0.001, and NS indicates P>0.05.

(S) and (R) refer to shoot and root tissue, respectively. ‘na’ indicates that the data were not available.

^b^NS-SNP: non-synonymous SNP. Variation based on information taken from RiceVarMap, http://ricevarmap.ncpgr.cn/.

^c^P-SNP: SNP within a 1 kb upstream promoter region. Variation based on information taken from RiceVarMap, http://ricevarmap.ncpgr.cn/.

**Fig 6:**
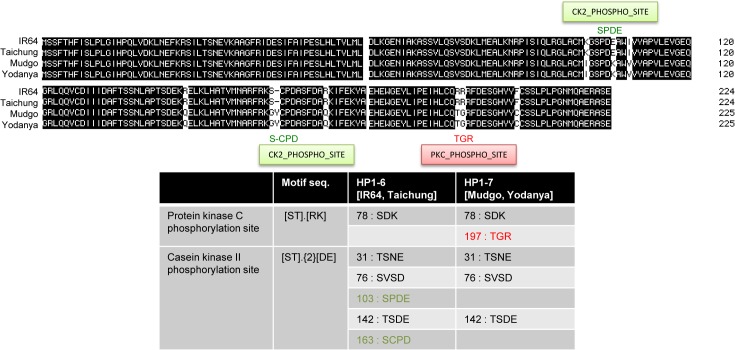
Protein sequence alignment of resequenced cDNA for candidate gene *PUE1-7* between haplotype groups HT1-6 and HT1-7. Top lanes show HT1-6 members (Taichung Native and IR64; bottom lanes show HT1-7 members (Mudgoe and Yodanyo). Haplotype-specific SNPs resulted in 10 amino acid changes, of which three affected kinase phosphorylation sites.

With four QTLs of interest, the overall number of candidate genes remains excessively large for a detailed investigation. We therefore limited ourselves to a more detailed analysis of only one QTL on chromosome 1 (peak at 7.275 Mb). This choice was based on the fact that this QTL was detected across experiments and that a minor haplotype increased PUE, which makes it of interest for breeding. To further examine SNP patterns at this QTL the 1479 accessions represented in the RiceVarMap database were screened for genotypes matching the 44 K-derived SNP pattern of the high-PUE haplotype 7 (HT1-7). The 108 accessions matching HT1-7 (haplotype frequency of 7.3%) are listed in S4 Table. Extracting all SNPs across the haplotype region and filtering ones specific for the 108 HT1-7 accessions produced a distinct SNP pattern, with an unusually high density of haplotype-specific SNPs around three genes, *PUE1-12* to *PUE1-14* (Fig 5B; S5 Table). Non-synonymous SNPs were predicted for *PUE1-1* (1), *PUE1-4* (1), *PUE1-7* (4), *PUE1-8* (1), *PUE1-10* (2), *PUE1-12* (14), *PUE1-13* (3) and *PUE1-14* (6). For *PUE1-7* these were confirmed by sequencing of the cDNA/gDNA (see Fig 6 for predicted protein sequence), demonstrating the power of haplotype-specific SNP prediction via SNP databases like RiceVarMap.

**Fig 7:**
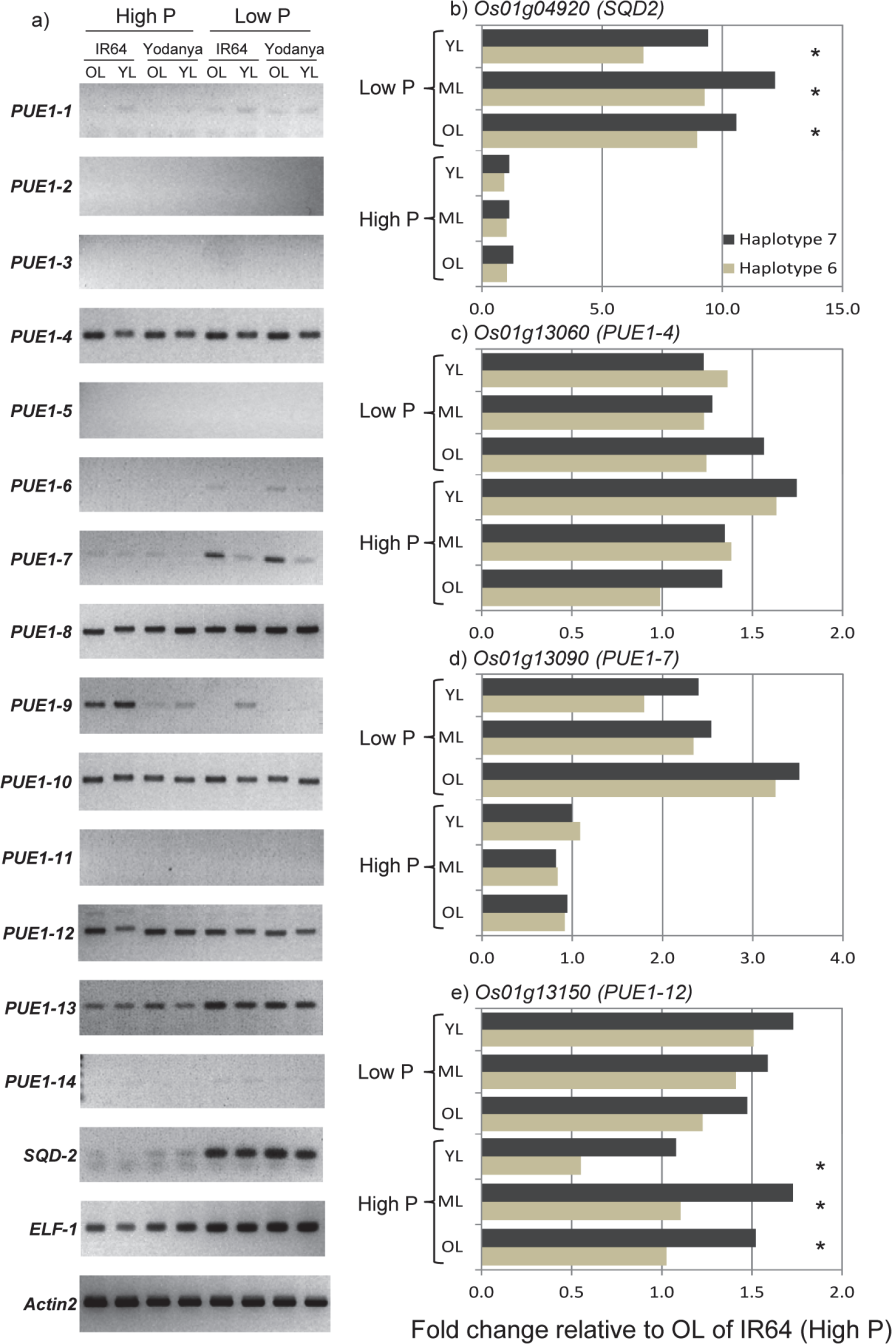
Gene expression analysis for candidate genes present within the PUE locus on chromosome 1 (7.24–7.34 Mb). Genotypes belong to two haplotype groups contrasting for PUE: HT1-6 with low PUE represented by IR64 and Taichung Native, and HT1-7 (high PUE) with representative Yodanya and Mudgo. RNA was sampled from P deficient plants (Low P) and P sufficient plants (High P). (a) Semiquantitative RT-PCR of all 14 genes within the locus. The known P-responsive gene SQD-2 was included as a control, as were two housekeeping genes. Gene expression was compared in old leaves (OL, beginning to senesce) and young leaves (YL, >50% developed). (b—e) qPCR for *SQD2* and the three most promising canditate genes. In addition to old and young leaves expression was determined in intermediate leaves (ML, typically the two leaves below the youngest fully expanded). Fold change for Fig 7b is expressed on a log scale.

Semi-quantitative PCR was performed for the known P-responsive gene *SQD2* in addition to all genes across the 7.22–7.36 Mb region for accessions IR64 (low PUE; HT1-6) and Yodanya (high PUE; HT1-7). Expression of SQD2 was strongly up-regulated by P deficiency (Fig 7A) and for gene *PUE1-7* a similar pattern was observed. This pattern was confirmed with qPCR: expression of *SQD2* increased about hundred-fold under P deficiency and was significantly higher in HT1-7 genotypes (Yodanya and Mudgo) compared to HT1-6 genotypes (IR64 and Taichung Native), irrespective of leaf age tested (Fig 7B). For *PUE1-7* P deficiency led to 2–3 fold up-regulation with highest expression detected in older leaves (Fig 7D), however, differences between haplotype groups were not significant. Other genes in the region were either not expressed or appeared to have similar expression levels across genotypes and P levels. This lack of regulation was confirmed by qPCR for gene *PUE1-4* (Fig 7C) and similar results were obtained for *PUE1-8*, *PUE1-10* and *PUE1-13* (data not shown). For *PUE1-12* about 50% higher expression levels were detected in HT1-7 genotypes compared to HT1-6 and this was limited to the +P treatment (Fig 7E). *PUE1-9* was only expressed in leaves of HT1-6 types under sufficient P supply and the lack of expression under P deficiency was confirmed by qPCR (data not shown).

*PUE1-7* encodes a nucleic acid binding protein (LOC_Os01g13090) with an RNA ligase/cyclic nucleotide phosphodiesterase domain (IPR009097) and a protein kinase A anchor protein nuclear localization signal domain (IPR019510). Sequencing the cDNA of the PUE1-7 alleles of both haplotype groups confirmed the presence of synonymous and non-synonymous SNPs predicted by RiceVarMap-based haplotype analysis. Collectively they resulted in haplotype-specific changes in protein sequence (Fig 6). Compared to HT1-7 the HT1-6 allele gained two Casein kinase II phosphorylation sites and lost one Protein kinase C phosphorylation site, possibly affecting responsiveness to kinase-mediated signal integration. Furthermore a highly conserved cysteine at AA 206 was converted to phenylalanine in the HT1-6 allele, possibly affecting tertiary structure due to impaired disulfide bridging.

*PUE1-12* (LOC_Os01g13150) is predicted to encode a metallo-beta-lactamase family protein with a ribonuclease Z domain (IPR013471). Sequencing of the cDNA/gDNA from the HT1-6 and HT1-7 haplotype groups confirmed rare synonymous and non-synonymous SNPs previously identified for the HT1-7 allele by RiceVarMap and suggested the presence of 14 amino acid changes between the HT1-6 and HT1-7 alleles (data not shown).

## Discussion

While there has been a strong push to breed P-efficient crop cultivars for environmental and economic reasons, progress has been limited and restricted to improvements in PAE [9]. However, enhanced PAE alone cannot improve the overall P efficiency of farming systems because higher P uptake leads to P mining in low-input systems, or increased P fertilizer requirements to replace P removed in harvested product in high-input systems. There is thus a growing recognition of the need to concurrently improve PUE in crop cultivars [6,7].

Several studies have reported genotypic variation for PUE across a range of crops and even mapped QTLs for PUE (reviewed in [20]). In all instances QTLs were mapped from experiments that concurrently assessed P uptake, and consequently, genotypic PUE estimates included components of true physiological PUE and artifacts resulting from differences in P uptake [15]. Given that variability for PAE is typically larger than for PUE [17] we proposed that these QTLs for PUE are actually indicative of P starvation in lines with poor P uptake, and are only ‘pseudo’ QTLs for PUE, affected by true QTLs for P uptake [7]. That none of these QTLs have been pursued in breeding programs further indicates their limited practical value.

In the present study, confounding effects of differences in P uptake were avoided by ensuring that all genotypes had near-equal P uptake, and P uptake was sufficient to eliminate any effects of seed P content on PUE (Table 2). Experiments were furthermore conducted under P-deficient conditions (mean total biomass reduced by 33% in Exp1 and 66% in Exp2) to ensure that loci identified are causally linked to P efficiency rather than to general vigor. However, for loci on chromosome 11 and 12 general vigor appears to have contributed to PUE (Table 3) as indicated by discernable (but non-significant) peaks at PUE loci. The most significant peak in Exp1 (chromosome 1, 7.27 Mb) did not coincide with general vigor in that experiment and therefore appears more PUE-specific despite showing a weak unspecific vigor peak in Exp2. Generally, unspecific vigor contributed to PUE to a higher degree in Exp2 (Tables 2 and 3) and this could be due to the more frequent P application that may have favored vigorous genotypes, whereas the one-time dose in Exp1 could have accentuated differences in the capacity for P redistribution and other more adaptive responses.

### Loci associated with PUE

The use of a broad association panel enabled the mapping of six loci associated with PUE. Two were linked to root biomass production under P deficiency, the remaining four were either shoot-specific or unspecific (total biomass). In combination with a subpopulation-specific analysis of linkage patterns around main peaks, it was possible to define linkage blocks surrounding peaks and thereby to delimit the most likely interval. Interval size ranged from 100 Kb to 150 Kb where linkage blocks and haplotypes were well defined. Thus, within the course of one study we identified loci and narrowed down their probable position to a level an order of magnitude smaller than possible with traditional QTL mapping.

Moreover, GWAS exploits a larger pool of genetic variability than bi-parental QTL mapping, thereby increasing the likelihood of detecting any possible adaptive mechanisms. Sub-population analyses also facilitated the detection of less frequent alleles that may segregate only in one or two sub-populations [21]. The haplotype analysis performed here confirms this point as a relatively rare *aus* haplotype (n = 8) on chromosome 11 was identified as being positive for high PUE. The most commonly used *aus* parent in bi-parental mapping populations, Kasalath, possesses the major haplotype and it would therefore not have been possible to identify the minor haplotype using Kasalath-derived mapping populations.

Analysis of the high PUE-associated region of chromosome 11 did not reveal high priority candidate genes, mainly due to insufficient functional annotation and low levels of trait-specific transcript responsiveness (S7 Table). Graphical haplotypes further revealed that the minor haplotype was partly defined by polymorphisms not captured by current SNP markers (Fig 3C). As for *Pup1*/*OsPSTOL*, it is possible that genes enhancing PUE are not present in the *japonica*-based reference genome. It would therefore be of interest to sequence the locus in accessions with the minor haplotype (e.g. Santhi Sufaid, DJ123, Coarse) in order to test whether larger chromosomal rearrangements similar to the *Pup1* locus [10,22] are present at this locus.

### Mechanisms linked to improved PUE and putative candidate genes

Metabolic pathways involved in remobilizing and recycling P from old to new tissues, the use of alternative glycolytic and electron transport pathways and replacement of phospholipids with sulfo- or galactolipids have all been implicated in the response of plants to P deprivation [23–26]. However, to the best of our knowledge, none of the numerous genes and metabolic pathways implicated in PUE have been shown to be causal mechanisms of genotypic P efficiency. Indeed, transcriptome studies on the model rice genotype ‘Nipponbare’ and a near-isogenic line containing the *Pup1* locus found that the typical transcriptional changes observed under P deficiency were as pronounced, or more pronounced, in the P-inefficient ‘Nipponbare’ [12]. The importance of distinguishing stress-response from stress-tolerance mechanisms/genes was recently confirmed in a study comparing sequenced mRNA transcripts in four rice cultivars of varying tolerance to P-deficiency [27]. One of these low-P responsive genes, *SQD2*, was used here as a ‘control’ in the gene expression analysis of candidate genes and we could confirm its responsiveness to P deficiency. Interestingly, it showed higher expression in accessions with superior PUE (HT1-7 group), suggesting that replacing phospholipids with sulfo- or galactolipids may have occurred at a higher rate in these efficient genotypes. Nevertheless *SQD2* did not co-localize with any identified QTL.

We used converging evidence from trait-associated nucleotide variation detected here with data from trait-associated expression profile databases and predicted gene function in our candidate gene approach. In respect to variation, the presence of a highly divergent region on chromosome 1 (7.22–7.34 Mb) with an enrichment of rare and haplotype-specific SNPs was most interesting. Among the 14 annotated genes in this region *PUE1-7* was found to be most responsive to P-availability (Fig 7) and was furthermore very closely associated with the peak SNP (id1005583) (Fig 5). Irrespective of the investigated genotypes/haplotypes, PUE1-7 displayed significant upregulation upon P-starvation, particularly in old leaves (Fig 7) suggesting functions in P remobilization. PUE1-7 shows homology to the N terminal nuclear localization signal domain of the A-kinase anchoring protein 7 (AKAP7_NLS), to the activating signal cointegrator 1 complex subunit 1(ASCC1) and to the RNA ligase/cyclic nucleotide phosphodiesterase (CPDase). AKAP7_NLS [28] and ASCC1 [29] are involved in protein scaffolding for transcriptional regulation, whereas CPDases have been implicated in nuclear tRNA metabolism [30]. It can therefore be assumed that *PUE1-7* localizes to the nucleus where it is either involved in transcriptional regulation, likely as part of a larger complex, or in RNA processing. Allelic variation of *PUE1-7* between HT1-6 and HT1-7 suggests that the respective proteins differ in structure and in domains for post-transcriptional modification (Fig 6). Consequently, differences in signal integration and/or protein-protein interactions could result in differential transcriptional modulations that are causal for the observed haplotype-associated differences in PUE.

Though not prominently transcriptionally responsive to P availability, several other candidate genes within the PUE1 region merit further consideration. Of particular interest are *PUE1-12* and *PUE1-13*, which were found to be differentially expressed by semiquantitative RT-PCR (Fig 7A) and for which RiceVarMap-based haplotype analysis suggested unusually high allelic variation (Fig 5). The former was confirmed for *PUE1-12* via qPCR (Fig 7E) and the latter was confirmed via cloning and sequencing for *PUE1-12* (data not shown). PUE1-12 is annotated as a putative zinc phosphodiesterase and shows homology to endoribonucleases involved in RNA metabolism. RNA can account for more than half of the non-storage P in plants [6], and therefore altered RNA turnover may have implications for PUE [31]. Fifteen rare haplotype-specific non-synonymous SNPs in the *PUE1-12* allele of HT1-7 translate to amino acid changes that might positively affect catalytic activity.

Without thorough transgenic validation studies, which are beyond the scope of this work, it will not be possible to determine which gene mutation is indeed causal for the observed haplotype-specific differences in PUE. However our data provides solid support for a high priority shortlist of candidate genes on chromosome 1 that merit further investigation.

### Potential utility of identified loci in rice breeding

Previous loci for PUE have been identified from screening studies where PUE was negatively associated with biomass as a result of poor P uptake of the more ‘efficient’ parent. Such QTL are of little practical value; to show promise for use in plant breeding PUE loci should be associated with higher biomass production, as observed in the present study. Thus, the loci identified in this study represent the first specific loci for PUE at the vegetative stage identified in any plant species that are not simply an artifact of low P uptake. By ruling out strong associations with height and stem weight (Table 2), the loci identified here were not simply associated with tall landrace accessions with a high proportion of stem, but rather, represent loci specific to PUE that have the capacity to enhance biomass production per unit of P uptake and therefore have utility in plant breeding.

That many of the modern cultivars produced in the early green revolution breeding programs (e.g. IR8, Taichung Native, Teqing, Zhenshan 2) lack all three positive haplotypes (Fig 4) supports the notion that breeding under favorable conditions has led to the loss of genes or alleles for adaptation to stressful environments in modern cultivars [8]. However, among the ‘-+-‘ group (Fig 4) are the hugely successful second generation green revolution varieties ‘IR36’ and ‘IR64’, which may at least partially explain why these cultivars have been adapted in less favorable environments as well. The absence in these mega-varieties of favorable haplotypes at the other two loci suggests real scope for introgressing the positive loci into elite rice cultivars to enhance P deficiency tolerance. Accessions in the ‘+++’ group include ‘Mudgo’, ‘Short Grain’ and ‘Yodanya’ which would represent promising donors in a breeding program.

The identification of the *PSTOL1* gene and the use of the *Pup1* locus in rice breeding programs has been the first step in improving the P efficiency of rice-based farming systems through plant breeding. Ideally, breeding programs would aim to combine the improved capacity to acquire P from the soil conferred by *Pup1*, with enhanced P utilization efficiency loci. Such ‘pyramiding’ of P efficiency loci should lead to the development of varieties that maintain high yields with reduced P fertilizer inputs while also having increasing yields on the millions of ha of rice area currently lacking a sufficient level of bioavailable P in soils.

## Materials and Methods

### Plant material

The broad rice association panel described in detail by Zhao *et al*. [18] was used in this study. Briefly, the panel comprises 413 diverse *Oryza sativa* accessions from 82 countries, representing the diversity of the primary gene pool of domesticated rice. Due to unsuccessful seed multiplication, poor germination or poor non-stress growth, only 292 accessions were included in experiment 1 (Exp1). For experiment 2 (Exp2) seed was imported into Japan and multiplied at the JIRCAS subtropical research station in Ishigaki/Okinawa. Because Exp1 revealed the highest degree of variability for PUE and the highest level of PUE within the *indica*, *aus* and tropical *japonica* subpopulations we only used accessions belonging to these three subpopulations in Exp2.

### Plant growth conditions

In Exp1, plants were grown in a screenhouse at IRRI, Los Banos, Philippines from January-March 2010. Average daily max/min temperatures were 34°C and 23°C, respectively. Seeds were germinated in petri dishes, raised on a floating mesh before being cultivated in Yoshida nutrient solution [32] in individual containers for 40 days (S1 Fig) following a modified version of the method of Rose *et al*. [15] that increased the one-off dose from 400 to 800 μg P in the low-P treatment. This increased P supply was meant to minimize effects of genotypic differences in seed P content. Plants in the high-P treatment received 4526 μg P total added in four installments (S1 Fig). Each genotype x P treatment combination was replicated four times.

Plants were watered daily with de-ionized water to maintain 250 mL of liquid in containers. Solution pH was buffered at pH 5.8 with MES and monitored weekly; necessary adjustments were made with 0.1N NaOH. Nutrients (other than P) were re-supplied as additional ‘shots’ to containers to achieve a 1 X Yoshida concentration in the 250 mL volume. The entire solution was replaced in the low-P treatment at 38 days after sowing (DAS) (S1 Fig). Colorimetric P measurements showed that added P was completely taken up within 5 days (S1 Table), so replacement of entire solutions at 38 DAS had no bearing on P uptake.

Exp2 was conducted in April-May 2012 in a heated glasshouse at JIRCAS, Tsukuba, Japan. Average daily max/min temperatures were 34°C and 25°C, respectively. The general procedure was as in Exp1, but rather than giving a one-time dose of P, low-P plants received a total of 1550 μg P added in six installments throughout the growing period and high-P plants received a total of 15.5 mg P in six installments (S1 Fig). Silicon was added weekly as K_2_O_3_Si, at 0.2 mM and entire solutions were replaced at 28 and 42 DAS.

To test how rapidly P is taken up by genotypes, P concentration remaining in the growing solution were measured in a preliminary experiment. Four days after adding 77.5 μg or 16.25 μg P, a 10mL aliquot was analyzed for P by inductively coupled plasma mass spectrometry (ICP-MS; ELAN DRC-e, PerkinElmer Inc., MA, USA) because colorimetric assays lacked the sensitivity to detect P in highly depleted solutions. Sampling was done 32- and 36 DAS for 21 genotypes. At 32 DAS average P concentrations detected were 0.05 μg L^-1^ P with a maximum of 3.7 μg L^-1^ P (S2 Fig). At 36 DAS most samples contained no detectable P and only three had remaining concentrations of around 3.1 μg L^-1^ P. These were different from accessions for which remnant P was detected at 32 DAS, indicating that genotype-specific effects probably did not exist. We conclude that more than 95% of added P was taken up within 4 days.

### Measurements

Both experiments were harvested at 50 DAS. Plant height, tiller number and root length were measured before shoots were severed from roots at the crown. Shoot and root material was oven-dried at 60°C for 4 days and weighed. Shoot and root material from a subset of genotypes was ground and a 0.5 g sample digested in a 3:1:1 nitric:perchloric:sulfuric acid mix. P concentrations were determined colorimetrically [33]. Root and shoot P contents (calculated by multiplying tissue P concentrations by biomass) were summed to obtain total P uptake, which was then compared to theoretical P uptake calculated by adding the known seed P content of each genotype to the amount of P added to each container.

### Genome wide association studies

The 44K rice SNP marker set was used for association analysis as described by Zhao *et al*. [18]. Associations between genotypic and phenotypic data were evaluated using TASSEL software version 3.0 [34]. Two approaches were followed in association analyses: General Linear Model (GLM) using phenotypes, genotypes and population structure as variables; and Mixed Linear Model (MLM) integrating a kinship matrix into phenotypes, genotypes and population structure. The kinship matrix was calculated from the 44K markers and QQ and Manhattan plots were generated to evaluate results. To establish the significance threshold we calculated a false discovery rate (FDR)-based Q value using the software QVALUE [35]. Based on the distribution of Q values obtained with MLM the false discovery rate remained stable and low up to a P value of 7.69E-04 but started to increase dramatically above 1.03E-03 (data not shown). A threshold P value of 7.0E-04 may thus be sufficient to avoid an excessive number of false positives. We typically considered peaks as relevant if they surpassed P < 1.0E-04 in at least one experiment. For significant loci, linkage disequilibrium (LD) analysis was performed on a sub-population basis, using the LD function in TASSEL. Linkage blocks were identified based on R^2^ values in linkage plots.

In order to investigate allelic variation associated with candidate genes (CGs), the comprehensive database RiceVarMap (http://ricevarmap.ncpgr.cn) was queried as follows. 44K SNP IDs of the haplotype defining SNPs were translated into RiceVarMap SNP IDs and all accessions that contained full high PUE-associated haplotypes across a linkage block were identified. All SNPs of the haplotype regions were extracted and filtered for subpopulation-specific allelic frequencies that matched the one found for the haplotype in question. These SNPs were then queried against all accessions carrying this haplotype to confirm their specificity. All unique/rare specific SNPS were then listed and checked for their position within gene loci and their effects (e.g. promoter region, intronic, non-synonymous).

### PCR conditions and DNA sequencing

Leaves of different developmental stages (old, medium, young) were sampled from 50 day old plants grown under low/high P conditions as described for Exp1. RNA was extracted and cDNA generated as per Pariasca-Tanaka et al. (2009). RT-PCR was performed using first-strand cDNA and ExTaq DNA polymerase (Takara, Japan) for 30 cycles (98°C, 10 sec; 60°C to 63°C, 10 sec; 72°C, 60 sec). For qPCR a Bio-Rad CFX96 Real-time PCR system and SYBR Premix ExTaq (Takara, Japan) was used under following conditions: 30 seconds at 95°C, followed by 40 cycles (95°C, 5 sec; 60 to 63°C, 20 sec). After 40 cycles, threshold cycle values were calculated automatically using default settings of the CFX Manager software (version 3.1; Bio-Rad). For re-sequencing of PUE1-7, cDNA obtained above was amplified by PrimeSTAR Max DNA Polymerase (Takara, Japan). PCR products were analyzed by direct-sequencing.

## Supporting Information

S1 Fig Diagram of experimental details for both hydroponic growth experiments. Plants were grown in Yoshida nutrient solution in individual containers under two P treatments: low-P and a +P control. Nutrients (except P) were supplied as ‘shots’ to containers as indicated by light blue ovals. Boxes around nutrient solution activities indicate entire solutions were replaced. P additions are indicated by dark rectangles (low-P) or unfilled rectangles (+P).
(PPTX)

S2 Fig P depletion from hydroponic solutions. A 10 mL volume was sampled from individual 1 L bottles four days after the addition of P (plants were 4 or 5 weeks old at 1st and 2nd sampling). P concentrations were analyzed by ICP-MS because colorimetric assays were not sensitive enough to detect any P.
(PPTX)

S3 Fig Relationship between estimated PUE and measured PUE for a subset of genotypes. Estimated PUE was based on P supply in nutrient solution and seeds whereas measured PUE was based on analysis of P concentrations in root and shoot tissue.
(PPTX)

S4 Fig Statistical Q-Q plots comparing expected *versus* actual probability values. (PPTX)

S5 Fig Linkage disequilibrium maps for PUE loci detected on chromosomes 1, 4, 11 and 13. (PPTX)

S1 Table Concentration of P (μM) remaining in low-P hydroponic solutions after 5 d of seedling growth. The initial concentration in low-P culture was 25.8 μM, indicating uptake of >95% of supplied P at this time.
(DOC)

S2 Table Mean PUE data of all accessions tested. (XLS)

S3 Table Location of significant peaks associated with plant height (Mb). (DOC)

S4 Table RiceVarMap accessions specific for haplotype HT1-7 on chromosome 1 or haplotype HT11-2 on chromosome 11. Data shown was used for variant analysis of candidate genes.
(DOC)

S5 Table Candidate genes at the highly significant peak on chromosome 1 (7.23–7.36 Mb). Reference columns show their regulation under P-deficiency in roots (R) and shoots (S), given as fold change in mRNA transcript abundance relative to P-sufficient plants (p>0.05, NS; p<0.05, *; p<0.01 **; p<0.001 ***). Transcripts not detected or reported are represented by ‘na’. Variation that was not determined is represented by ‘ND’.
(DOC)

S6 Table Candidate genes at the highly significant peak on chromosome 4 (17.7–17.85 Mb). Reference columns show their regulation under P-deficiency in roots (R) and shoots (S), given as fold change in mRNA transcript abundance relative to P-sufficient plants (p>0.05, NS; p<0.05, *; p<0.01 **; p<0.001 ***). Transcripts not detected or reported are represented by ‘na’. Variation that was not determined is represented by ‘ND’.
(DOC)

S7 Table Candidate genes at the highly significant peak on chromosome 11 (16.63–16.75 Mb). Reference columns show their regulation under P-deficiency in roots (R) and shoots (S), given as fold change in mRNA transcript abundance relative to P-sufficient plants (p>0.05, NS; p<0.05, *; p<0.01 **; p<0.001 ***). Transcripts not detected or reported are represented by ‘na’. Variation that was not determined is represented by ‘ND’.
(DOC)

S8 Table Candidate genes at the highly significant peak on chromosome 12 (17.7–17.85 Mb). Reference columns show their regulation under P-deficiency in roots (R) and shoots (S), given as fold change in mRNA transcript abundance relative to P-sufficient plants (p>0.05, NS; p<0.05, *; p<0.01 **; p<0.001 ***). Transcripts not detected or reported are represented by ‘na’. Variation that was not determined is represented by ‘ND’.
(DOC)
