# Supplementary material for: Unmasking Novel Loci for Internal Phosphorus Utilization Efficiency in Rice Germplasm through Genome-Wide Association Analysis
Source: PLoS One. 2015 Apr 29;10(4):e0124215. doi: 10.1371/journal.pone.0124215 (PMC4414551; doi:10.1371/journal.pone.0124215)
Supplement: S1 Fig — Plants were grown in Yoshida nutrient solution in individual containers under two P treatments: low-P and a +P control. Nutrients (except P) were supplied as ‘shots’ to containers as indicated by light blue ovals. Boxes around nutrient solution activities indicate entire solutions were replaced. P additions are indicated by dark rectangles (low-P) or unfilled rectangles (+P). (PPTX) [file pone.0124215.s001.pptx]

## Slide 1
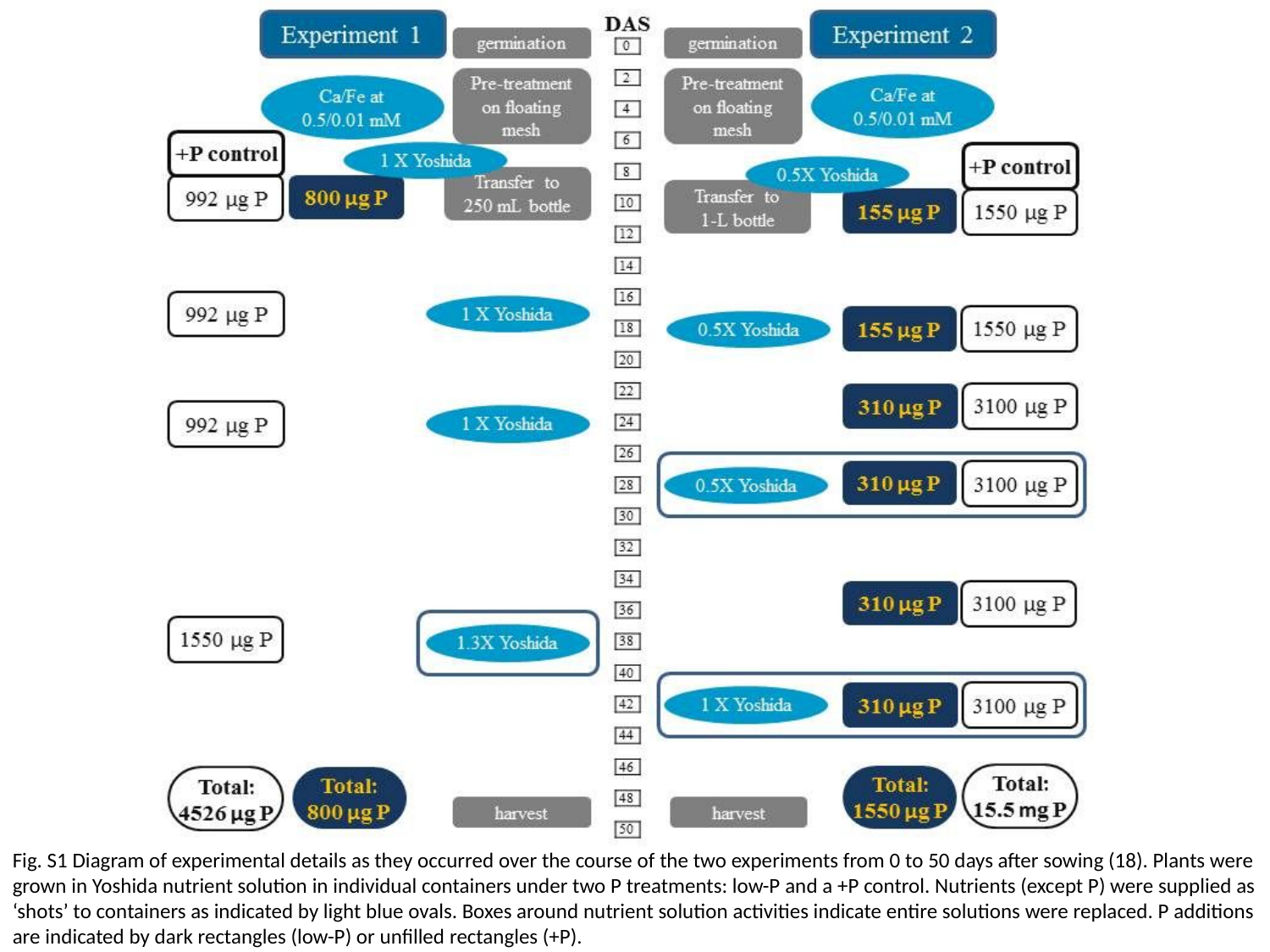

Fig. S1 Diagram of experimental details as they occurred over the course of the two experiments from 0 to 50 days after sowing (18). Plants were grown in Yoshida nutrient solution in individual containers under two P treatments: low-P and a +P control. Nutrients (except P) were supplied as ‘shots’ to containers as indicated by light blue ovals. Boxes around nutrient solution activities indicate entire solutions were replaced. P additions are indicated by dark rectangles (low-P) or unfilled rectangles (+P).
