# Supplementary material for: Unmasking Novel Loci for Internal Phosphorus Utilization Efficiency in Rice Germplasm through Genome-Wide Association Analysis
Source: PLoS One. 2015 Apr 29;10(4):e0124215. doi: 10.1371/journal.pone.0124215 (PMC4414551; doi:10.1371/journal.pone.0124215)
Supplement: S2 Fig — A 10 mL volume was sampled from individual 1 L bottles four days after the addition of P (plants were 4 or 5 weeks old at 1st and 2nd sampling). P concentrations were analyzed by ICP-MS because colorimetric assays were not sensitive enough to detect any P. (PPTX) [file pone.0124215.s002.pptx]

## Slide 1
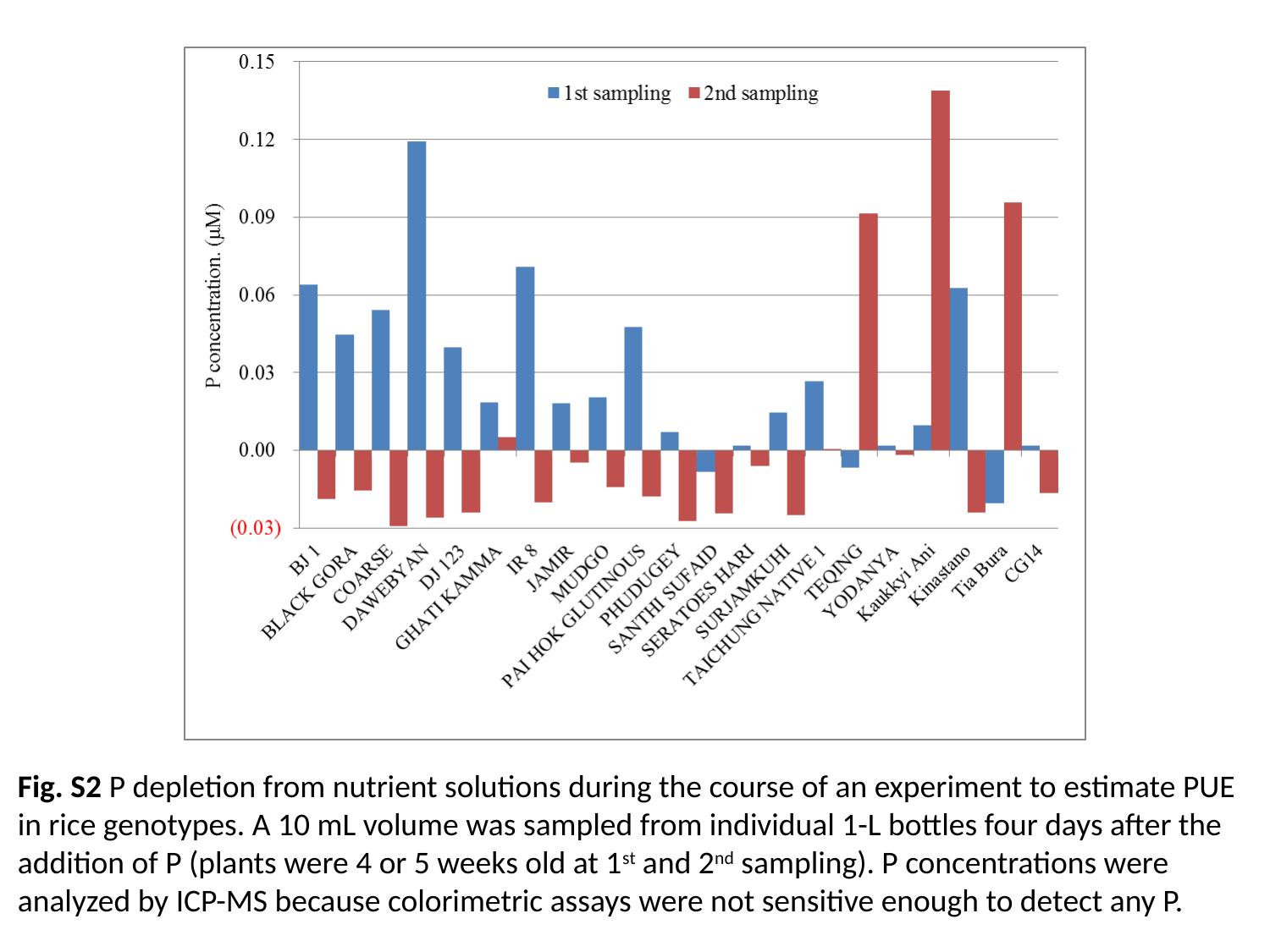

Fig. S2 P depletion from nutrient solutions during the course of an experiment to estimate PUE in rice genotypes. A 10 mL volume was sampled from individual 1-L bottles four days after the addition of P (plants were 4 or 5 weeks old at 1st and 2nd sampling). P concentrations were analyzed by ICP-MS because colorimetric assays were not sensitive enough to detect any P.
