# Supplementary material for: Unmasking Novel Loci for Internal Phosphorus Utilization Efficiency in Rice Germplasm through Genome-Wide Association Analysis
Source: PLoS One. 2015 Apr 29;10(4):e0124215. doi: 10.1371/journal.pone.0124215 (PMC4414551; doi:10.1371/journal.pone.0124215)
Supplement: S3 Fig — Estimated PUE was based on P supply in nutrient solution and seeds whereas measured PUE was based on analysis of P concentrations in root and shoot tissue. (PPTX) [file pone.0124215.s003.pptx]

## Slide 1
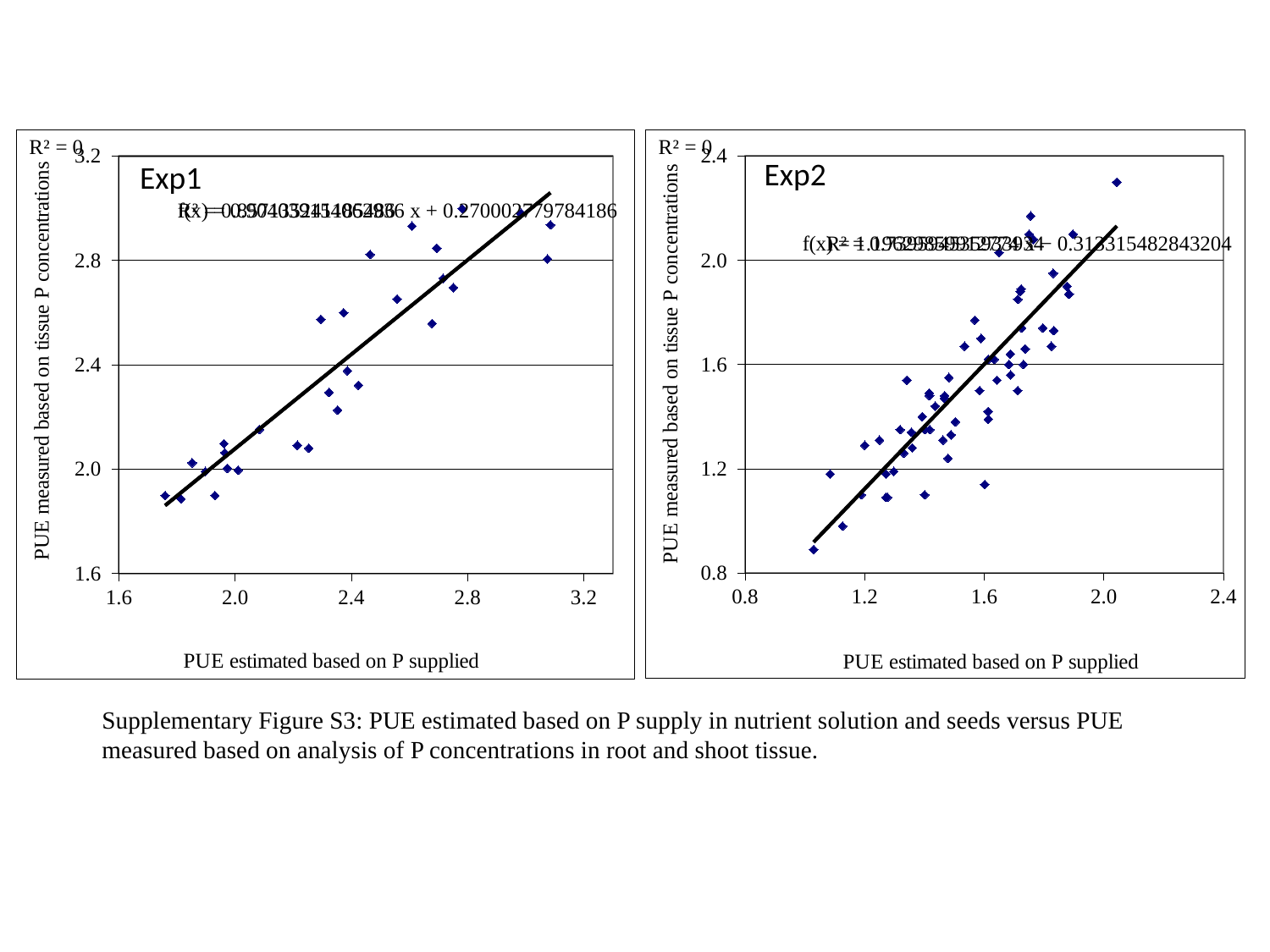

### Chart
| Category | | Peff90% |
|---|---|---|
### Chart
| Category | | #REF! |
|---|---|---|Exp2
Exp1
Supplementary Figure S3: PUE estimated based on P supply in nutrient solution and seeds versus PUE measured based on analysis of P concentrations in root and shoot tissue.
