# Supplementary figures and images for: Unmasking Novel Loci for Internal Phosphorus Utilization Efficiency in Rice Germplasm through Genome-Wide Association Analysis
Source: PLoS One. 2015 Apr 29;10(4):e0124215. doi: 10.1371/journal.pone.0124215 (PMC4414551; doi:10.1371/journal.pone.0124215)

## Slide 1
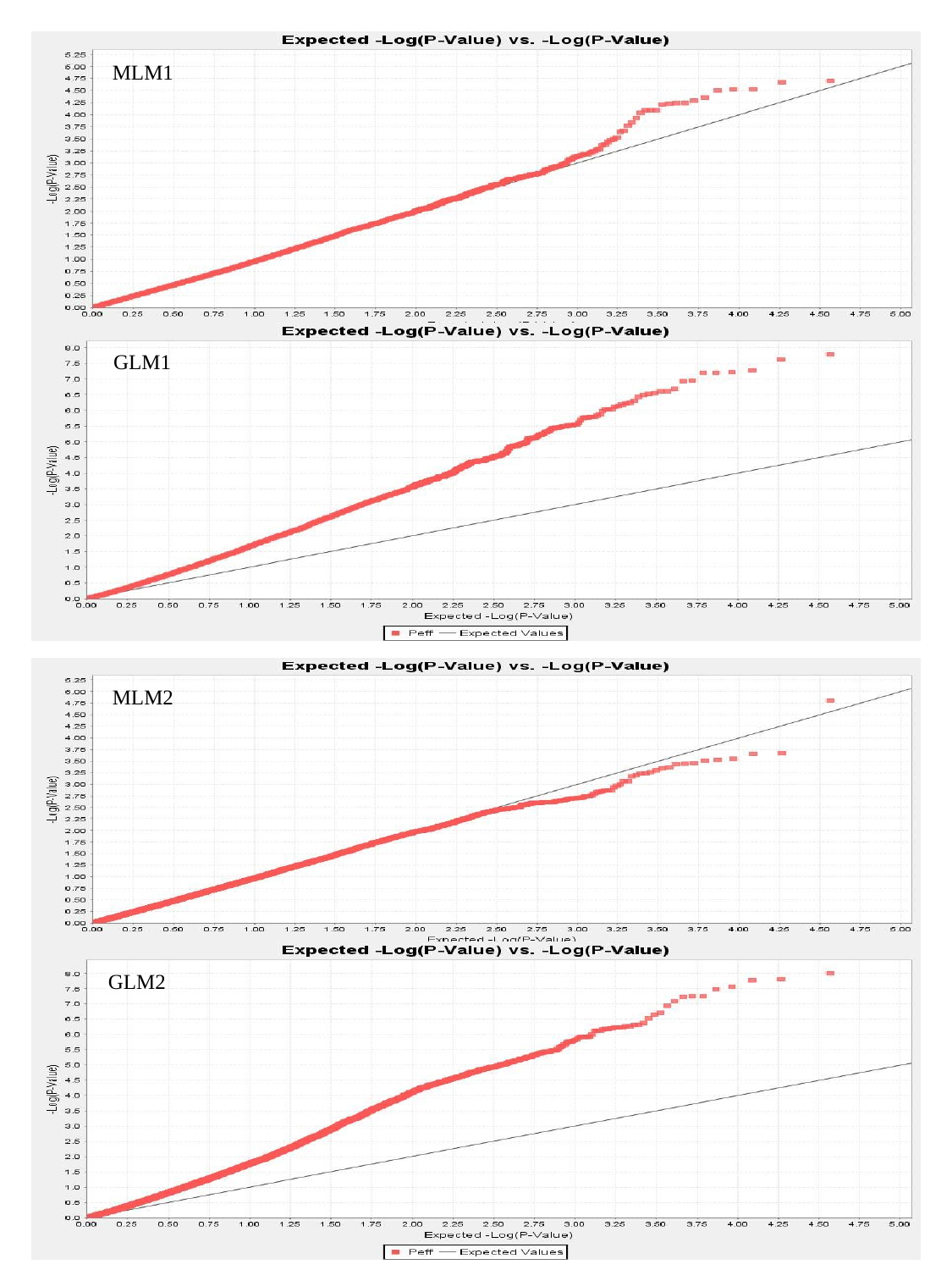

MLM1
GLM1
MLM2
GLM2

Supplement: S4 Fig — (PPTX) [file pone.0124215.s004.pptx]
