# Supplementary material for: Unmasking Novel Loci for Internal Phosphorus Utilization Efficiency in Rice Germplasm through Genome-Wide Association Analysis
Source: PLoS One. 2015 Apr 29;10(4):e0124215. doi: 10.1371/journal.pone.0124215 (PMC4414551; doi:10.1371/journal.pone.0124215)
Supplement: S5 Fig — (PPTX) [file pone.0124215.s005.pptx]

## Slide 1
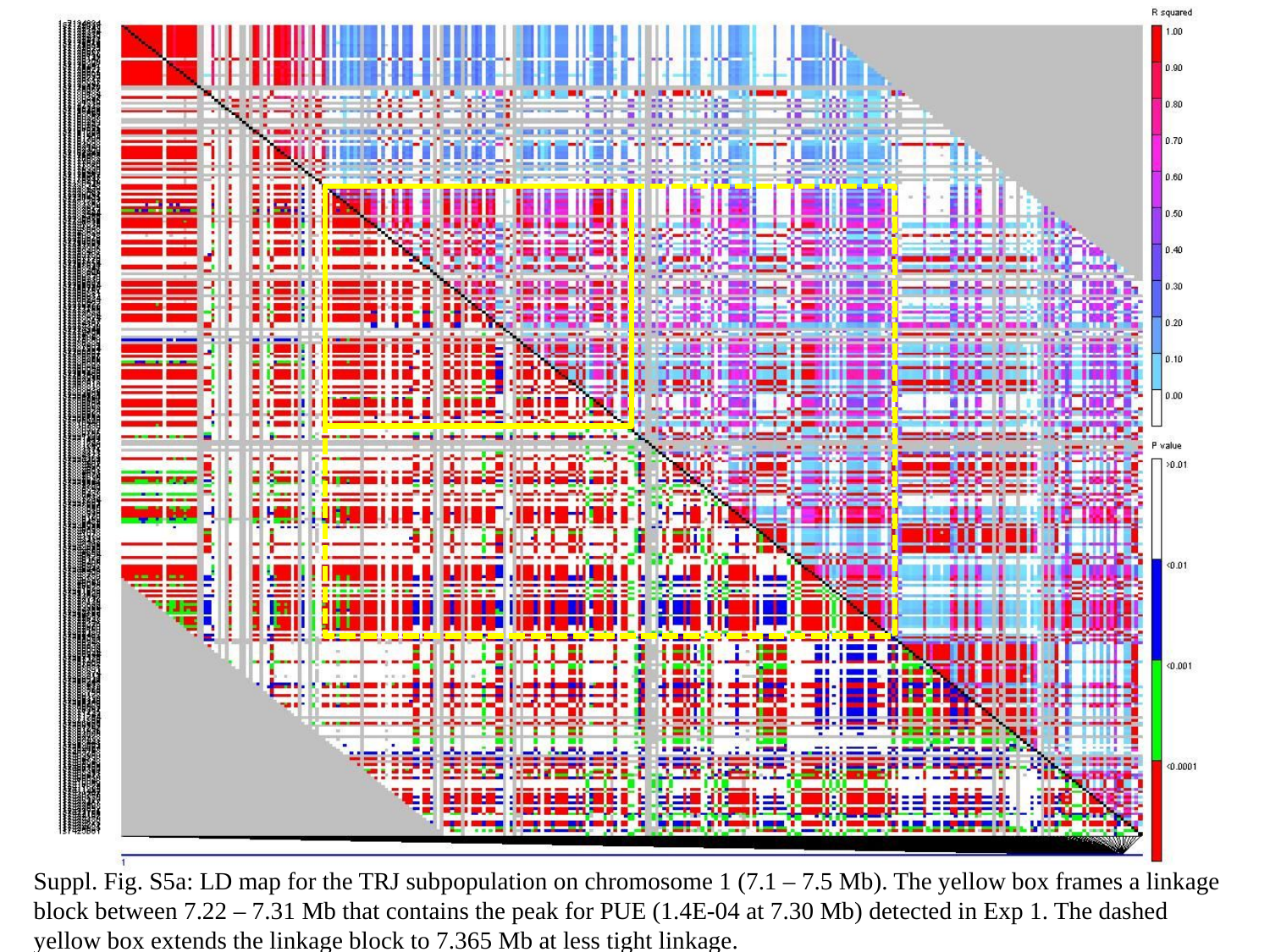

Suppl. Fig. S5a: LD map for the TRJ subpopulation on chromosome 1 (7.1 – 7.5 Mb). The yellow box frames a linkage block between 7.22 – 7.31 Mb that contains the peak for PUE (1.4E-04 at 7.30 Mb) detected in Exp 1. The dashed yellow box extends the linkage block to 7.365 Mb at less tight linkage.

## Slide 2
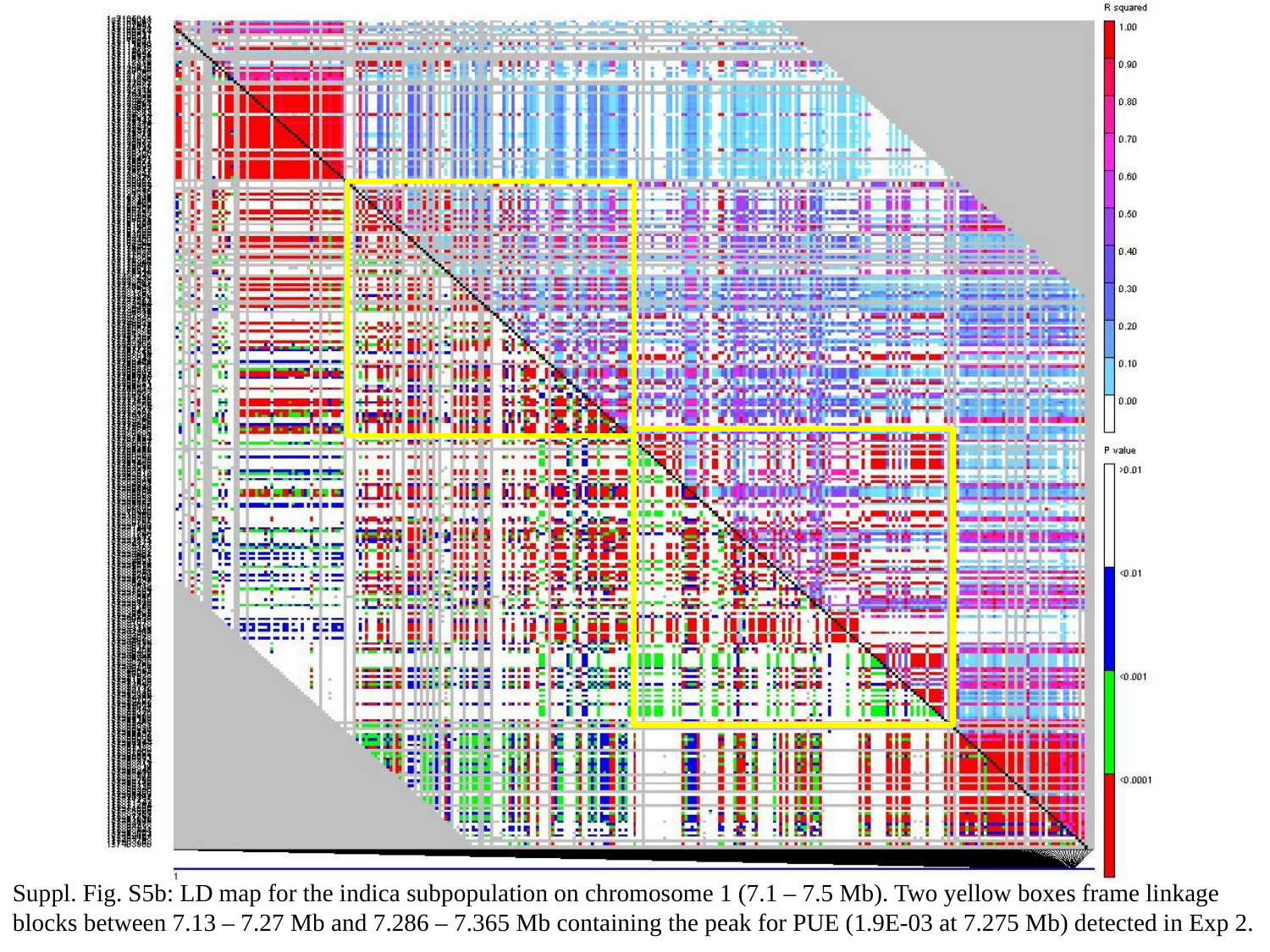

Suppl. Fig. S5b: LD map for the indica subpopulation on chromosome 1 (7.1 – 7.5 Mb). Two yellow boxes frame linkage blocks between 7.13 – 7.27 Mb and 7.286 – 7.365 Mb containing the peak for PUE (1.9E-03 at 7.275 Mb) detected in Exp 2.

## Slide 3
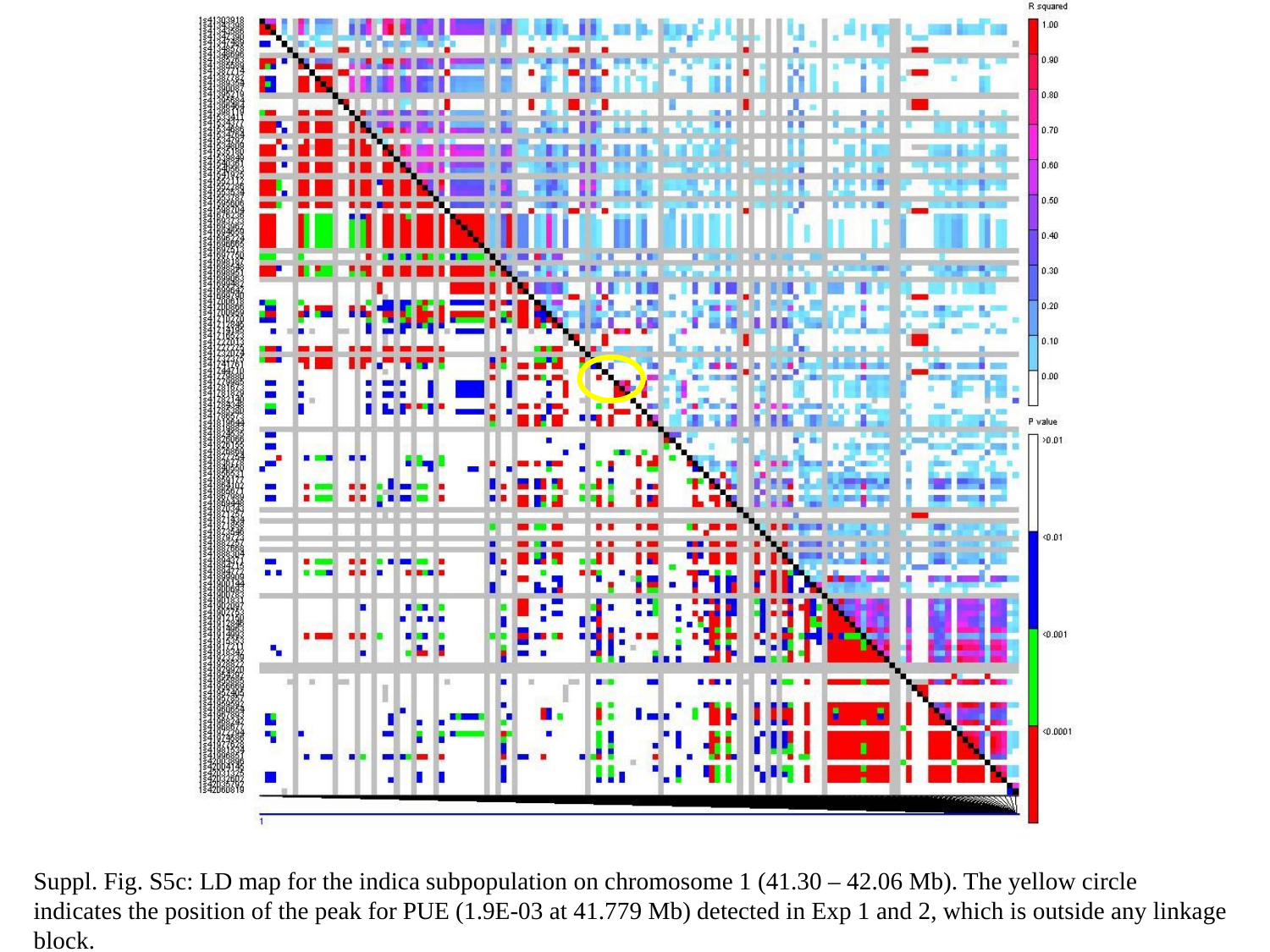

Suppl. Fig. S5c: LD map for the indica subpopulation on chromosome 1 (41.30 – 42.06 Mb). The yellow circle indicates the position of the peak for PUE (1.9E-03 at 41.779 Mb) detected in Exp 1 and 2, which is outside any linkage block.

## Slide 4
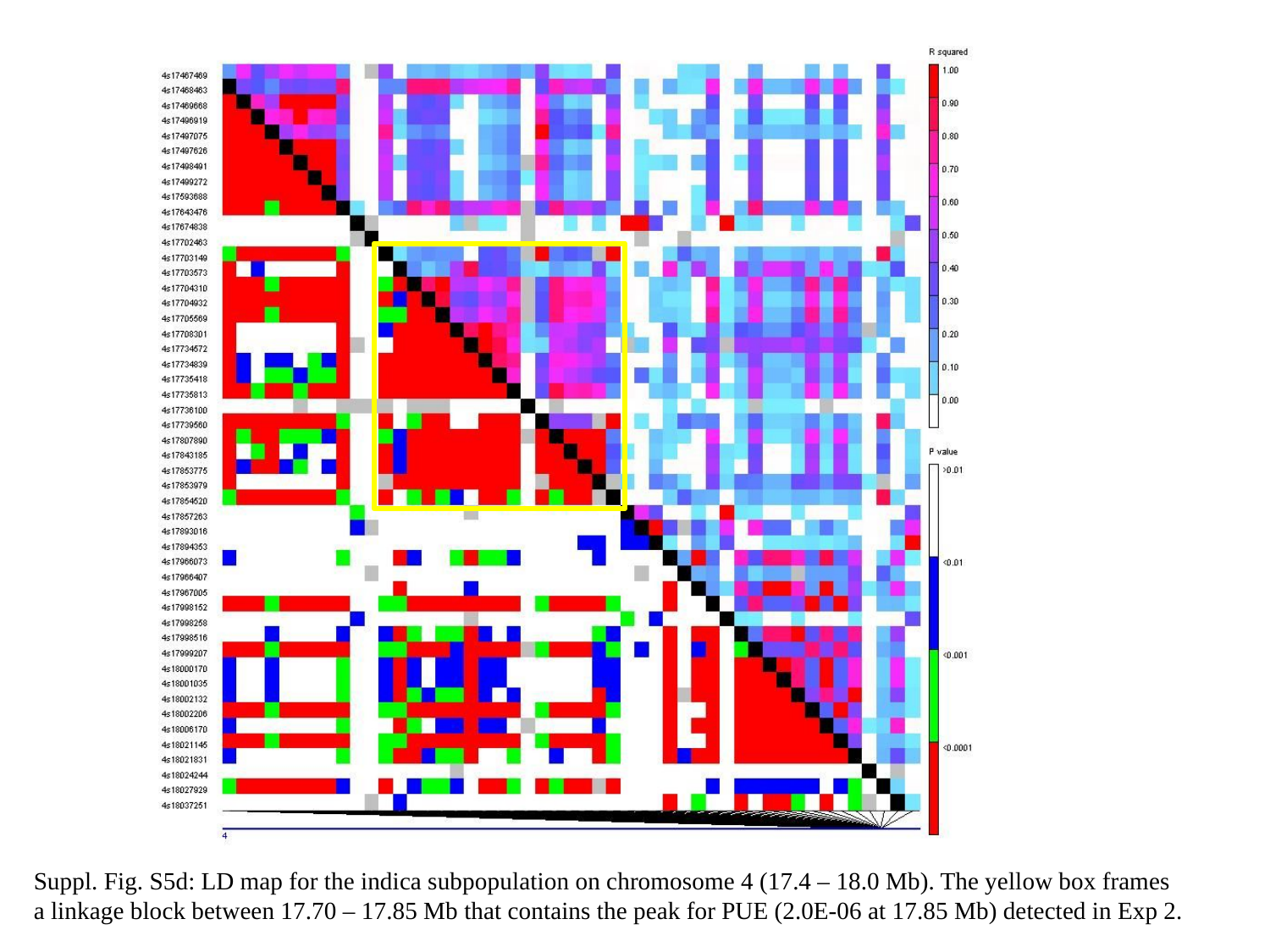

Suppl. Fig. S5d: LD map for the indica subpopulation on chromosome 4 (17.4 – 18.0 Mb). The yellow box frames a linkage block between 17.70 – 17.85 Mb that contains the peak for PUE (2.0E-06 at 17.85 Mb) detected in Exp 2.

## Slide 5
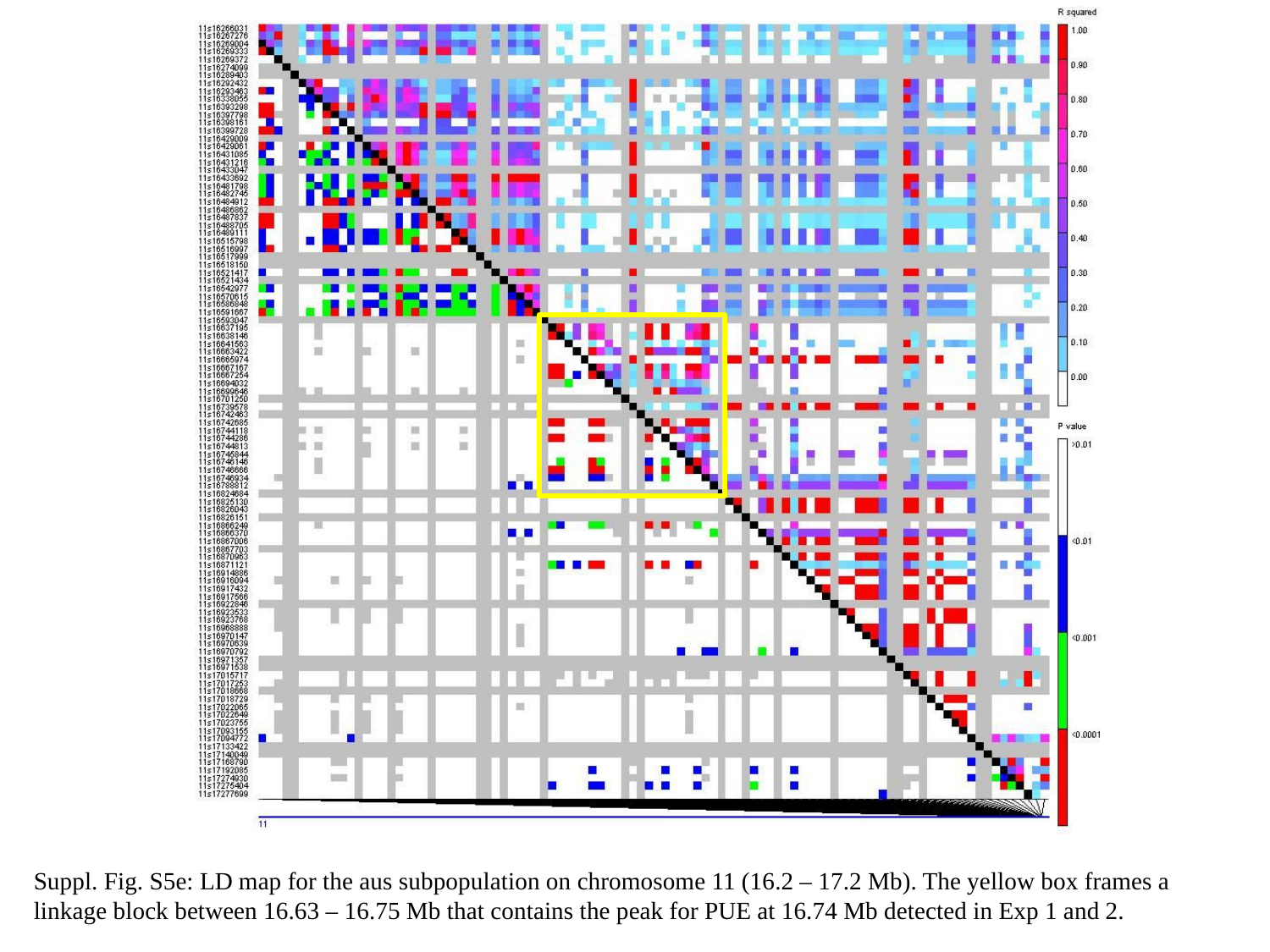

Suppl. Fig. S5e: LD map for the aus subpopulation on chromosome 11 (16.2 – 17.2 Mb). The yellow box frames a linkage block between 16.63 – 16.75 Mb that contains the peak for PUE at 16.74 Mb detected in Exp 1 and 2.

## Slide 6
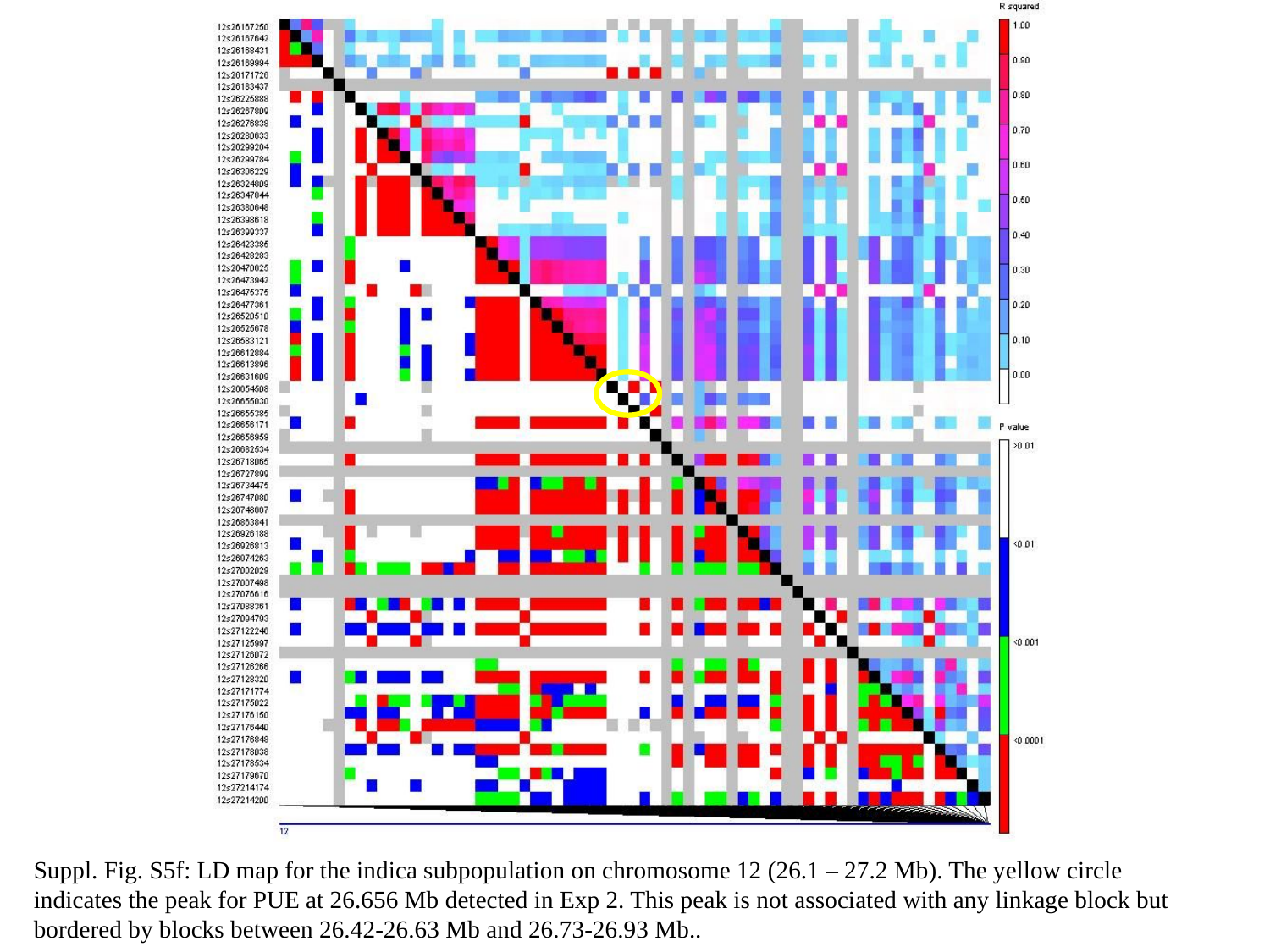

Suppl. Fig. S5f: LD map for the indica subpopulation on chromosome 12 (26.1 – 27.2 Mb). The yellow circle indicates the peak for PUE at 26.656 Mb detected in Exp 2. This peak is not associated with any linkage block but bordered by blocks between 26.42-26.63 Mb and 26.73-26.93 Mb..
