# Supplementary material for: Unmasking Novel Loci for Internal Phosphorus Utilization Efficiency in Rice Germplasm through Genome-Wide Association Analysis
Source: PLoS One. 2015 Apr 29;10(4):e0124215. doi: 10.1371/journal.pone.0124215 (PMC4414551; doi:10.1371/journal.pone.0124215)
Supplement: S1 Table — The initial concentration in low-P culture was 25.8 μM, indicating uptake of >95% of supplied P at this time. (DOC) [file pone.0124215.s006.doc]

**Table S1.** Concentration of P (µM) remaining in low-P hydroponic solutions after 5 d of seedling growth. The initial concentration in low-P culture was 25.8 µM, indicating uptake of >95% of supplied P at this time.

| Genotype | Replicate 1 | Replicate 2 | Replicate 3 |
| --- | --- | --- | --- |
| Bellardone | < 0.5 | < 0.5 | < 0.5 |
| C1-6-5-3 | < 0.5 | < 0.5 | < 0.5 |
| Jalmagna | < 0.5 | < 0.5 | < 0.5 |
| Nerica 9 | < 0.5 | < 0.5 | < 0.5 |
| Nerica 10 | < 0.5 | < 0.5 | < 0.5 |
| CG14 | < 0.5 | < 0.5 | < 0.5 |
| DNJ 140 | < 0.5 | 0.92 | < 0.5 |
| Ai-Chiao-Hong | < 0.5 | < 0.5 | < 0.5 |
| Dular | < 0.5 | < 0.5 | < 0.5 |
| WAB181 | < 0.5 | < 0.5 | < 0.5 |
| Nerica 17 | < 0.5 | < 0.5 | < 0.5 |
| Nerica 1 | < 0.5 | < 0.5 | < 0.5 |
| Nerica 18 | < 0.5 | < 0.5 | < 0.5 |
| Creole | < 0.5 | < 0.5 | < 0.5 |
| Teqing | < 0.5 | < 0.5 | < 0.5 |
| Nipponbare | < 0.5 | < 0.5 | 0.75 |
