# Supplementary material for: Unmasking Novel Loci for Internal Phosphorus Utilization Efficiency in Rice Germplasm through Genome-Wide Association Analysis
Source: PLoS One. 2015 Apr 29;10(4):e0124215. doi: 10.1371/journal.pone.0124215 (PMC4414551; doi:10.1371/journal.pone.0124215)
Supplement: S4 Table — Data shown was used for variant analysis of candidate genes. (DOC) [file pone.0124215.s009.doc]

**Table S4.** RiceVarMap accessions specific for haplotype HT1-7 on chromosome 1 or haplotype HT11-2 on chromosome 11, used for variant analysis of candidate genes

| Cultivar Name | Cultivar ID | Subpopulation | Location | Longitude | Latitude | Haplotype |
| --- | --- | --- | --- | --- | --- | --- |
| Shufeng101 | C036 | IndI | Sichuan | 30.39 | 104.05 | HT1-7 |
| Sanbaili | C055 | IndI | Hunan | 28.11 | 113 | HT1-7 |
| Menjiagao1 | C094 | Ind | Hainan | 20.2 | 110.3 | HT1-7 |
| Jinxibai-1 | C096 | Ind | Jiangxi | 28.41 | 115.52 | HT1-7 |
| Jinbaoyin | C097 | Ind | Fujian | 26.05 | 119.18 | HT1-7 |
| Hongainuo | C098 | Ind | Guangxi | 22.48 | 108.2 | HT1-7 |
| WH099 | C099 | Ind | Guangxi | 22.48 | 108.2 | HT1-7 |
| Taidongludao | C123 | TrJ | Taiwan | 25.03 | 121.31 | HT1-7 |
| Zegu | C136 | IndI | Guizhou | 26.35 | 106.42 | HT1-7 |
| Younian | C191 | IndI | Guizhou | 26.35 | 106.42 | HT1-7 |
| Wukezhan | C192 | Ind | Fujian | 26.05 | 119.18 | HT1-7 |
| Zinuo | C198 | IndI | Yunnan | 25.04 | 102.41 | HT1-7 |
| Yizhixiang | C201 | Ind | Fujian | 26.05 | 119.18 | HT1-7 |
| Xiaobaimi | C202 | IndI | Guizhou | 26.35 | 106.42 | HT1-7 |
| Te-tep | GP41 | Ind | Vietnam | NA | NA | HT1-7 |
| Bashuonuo | GP502 | TrJ | Japan | NA | NA | HT1-7 |
| Lunonglinnuo-1 | GP504 | TrJ | Japan | NA | NA | HT1-7 |
| Lunonglinnuo-12 | GP505 | TrJ | Japan | NA | NA | HT1-7 |
| Shijie-1 | GP561 | TrJ | Japan | NA | NA | HT1-7 |
| Shuifunuo | GP596 | Jap | Japan | NA | NA | HT1-7 |
| Abang Busur | GP628 | Ind | Indonesia | NA | NA | HT1-7 |
| Shanglanxiannuo-19 | HP120 | IndI | China | 18.25 | 109.5 | HT1-7 |
| Zhuxuenuo | HP121 | Ind | China | 23.08 | 113.14 | HT1-7 |
| Hangxiandahezi | HP174 | Ind | China | 25.85 | 114.02 | HT1-7 |
| Ningdexian | HP177 | Ind | China | 27.57 | 119.7 | HT1-7 |
| Honggu-2 | HP182 | Ind | China | 26.84 | 116.32 | HT1-7 |
| Dayeliu | HP195 | IndI | China | 30.16 | 120.1 | HT1-7 |
| Huazhan | HP201 | Ind | China | 25 | 117.53 | HT1-7 |
| Erdonghe-1 | HP206 | IndI | China | 27.05 | 118.32 | HT1-7 |
| Mazimang | HP207 | Ind | China | 27.76 | 118.02 | HT1-7 |
| Leigongman | HP213 | Ind | China | 25 | 117.53 | HT1-7 |
| Hongmiguyoumang | HP215 | Ind | China | 26.85 | 116.82 | HT1-7 |
| Liuzhuzhan | HP218 | Ind | China | 22.87 | 110.53 | HT1-7 |
| Qihe | HP230 | Ind | China | 22.69 | 109.2 | HT1-7 |
| Jiangzhan | HP231 | Ind | China | 22.36 | 106.84 | HT1-7 |
| Yahezhong | HP237 | Ind | China | 26.32 | 115.33 | HT1-7 |
| Simaobai | HP238 | Ind | China | 27.44 | 115.82 | HT1-7 |
| Datiezao | HP239 | Ind | China | 27.12 | 119.36 | HT1-7 |
| Handao | HP243 | IndII | China | 24.77 | 112.4 | HT1-7 |
| Changainuo | HP250 | IndI | China | 27.92 | 110.57 | HT1-7 |
| Changruangu | HP264 | IndI | China | 25.67 | 104.24 | HT1-7 |

**Table S4.** Continued; RiceVarMap accessions specific for haplotype HT1-7 on chromosome 1 or haplotype HT11-2 on chromosome 11, used for variant analysis of candidate genes

| Cultivar Name | Cultivar ID | Subpopulation | Location | Longitude | Latitude | Haplotype |
| --- | --- | --- | --- | --- | --- | --- |
| Hongjiaozhan | HP287 | Ind | China | 28.96 | 105.46 | HT1-7 |
| Hongshannuo | HP290 | Ind | China | 23.62 | 105.6 | HT1-7 |
| Baiguxiang | HP297 | Ind | China | 24.59 | 112.07 | HT1-7 |
| Benguzhong | HP302 | Ind | China | 22.69 | 109.2 | HT1-7 |
| Xibengu | HP303 | Ind | China | 24.67 | 109.24 | HT1-7 |
| Honghandao-2 | HP305 | Ind | China | 23.15 | 106.41 | HT1-7 |
| Dahonggu | HP311 | IndI | China | 34.79 | 116.57 | HT1-7 |
| Honghandao-1 | HP319 | IndI | China | 32.03 | 118.46 | HT1-7 |
| Maguhongmi | HP327 | Ind | China | 23.4 | 113.19 | HT1-7 |
| Hongmimozhan | HP332 | IndI | China | 28.37 | 112.8 | HT1-7 |
| Yangmianzao | HP333 | IndI | China | 28.16 | 113.63 | HT1-7 |
| Nanxiangzao | HP336 | IndI | China | 29.41 | 112.16 | HT1-7 |
| Bodao | HP339 | IndI | China | 19.52 | 109.57 | HT1-7 |
| Wenchangzhan | HP342 | Ind | China | 19.91 | 109.69 | HT1-7 |
| Qiwei | HP347 | Ind | China | 21.52 | 110.99 | HT1-7 |
| Heinuo | HP349 | Ind | China | 22.16 | 111.78 | HT1-7 |
| Gouzaizhan | HP350 | Ind | China | 21.71 | 112.76 | HT1-7 |
| Dajinggu | HP357 | Ind | China | 23.7 | 113.01 | HT1-7 |
| Magu-3 | HP397 | IndI | China | 28.38 | 111.2 | HT1-7 |
| Hongzuigu | HP412 | Ind | China | 22.69 | 109.2 | HT1-7 |
| Dazhangu | HP413 | IndI | China | 23.08 | 107.12 | HT1-7 |
| Baipigu | HP415 | IndI | China | 22.85 | 107.21 | HT1-7 |
| Dashuihe | HP417 | Ind | China | 22.64 | 110.14 | HT1-7 |
| Haonuoliang | HP422 | Ind | China | 21.95 | 100.5 | HT1-7 |
| Liutiaozi | HP440 | Ind | China | 26.46 | 116 | HT1-7 |
| Baimiyahzouzao | HP444 | IndI | China | 27.44 | 112.18 | HT1-7 |
| Baimiyahzouzao | HP444 | IndI | China | 27.44 | 112.18 | HT1-7 |
| Honghanzhangu | HP445 | Ind | China | 25.44 | 105.18 | HT1-7 |
| Zhuxizao | HP450 | Ind | China | 27.76 | 118.02 | HT1-7 |
| Heiqunzhan | HP451 | IndI | China | 21.68 | 110.88 | HT1-7 |
| Chuanda-1 | HP452 | IndI | China | 30.99 | 104.25 | HT1-7 |
| Ershiyihao | HP455 | Ind | China | 18.64 | 109.7 | HT1-7 |
| Zaogu-E | HP458 | IndI | China | 23.08 | 113.14 | HT1-7 |
| Hongmiqinghezhan | HP474 | IndI | China | 30.35 | 114.17 | HT1-7 |
| Jiangxiwan | HP476 | Ind | China | 24.55 | 116.1 | HT1-7 |
| Baizaogu | HP480 | IndI | China | 30.35 | 114.17 | HT1-7 |
| Jinzhan | HP486 | IndI | China | 27.92 | 109.43 | HT1-7 |
| Maweizhan | HP494 | Ind | China | 25.08 | 113.91 | HT1-7 |
| Hongmidongzhan | HP503 | Ind | China | 26.41 | 117.77 | HT1-7 |
| Yizhixiang | HP523 | Ind | China | 24.51 | 117.35 | HT1-7 |
| Changjingchi | HP524 | IndI | China | 25.12 | 117.01 | HT1-7 |

**Table S4.** Continued; RiceVarMap accessions specific for haplotype HT1-7 on chromosome 1 or haplotype HT11-2 on chromosome 11, used for variant analysis of candidate genes

| Cultivar Name | Cultivar ID | Subpopulation | Location | Longitude | Latitude | Haplotype |
| --- | --- | --- | --- | --- | --- | --- |
| Haoanxiu | HP529 | Ind | China | 21.95 | 100.5 | HT1-7 |
| Hongxinnuo | HP530 | Ind | China | 24.82 | 99.61 | HT1-7 |
| Guazizhan | HP537 | IndI | China | 30.4 | 104.04 | HT1-7 |
| Jiaozhan | HP547 | Ind | China | 23.38 | 110.07 | HT1-7 |
| Lengshuizi(Yehe) | HP555 | Ind | China | 26.46 | 116 | HT1-7 |
| Dahongjiao-3 | HP597 | IndI | China | 24.44 | 100.12 | HT1-7 |
| Haonuobi | HP607 | Ind | China | 21.95 | 100.5 | HT1-7 |
| Haonuobixian | HP608 | Ind | China | 24.69 | 97.93 | HT1-7 |
| Erzaogu | HP61 | IndI | China | 25.04 | 102.42 | HT1-7 |
| Nantexuan | HP613 | Ind | China | 19.36 | 110.1 | HT1-7 |
| Huangkejin | HP76 | IndI | China | 28.4 | 115.55 | HT1-7 |
| BERLIN | W009 | Ind | Costa Rica | 10 | -84 | HT1-7 |
| Thang 10 | W030 | Ind | Vietnam | 21.03 | 105.85 | HT1-7 |
| Toga | W054 | Ind | India | 28.6 | 77.2 | HT1-7 |
| Kao Chio Lin Chou | W058 | IndI | Taiwan | 25 | 121.7 | HT1-7 |
| AKP 4 | W071 | Ind | India | 28.6 | 77.2 | HT1-7 |
| TAINUNG 45 | W074 | Ind | Taiwan | 25.02 | 121.38 | HT1-7 |
| Sereno | W111 | Ind | Jamaica | 17.58 | -76.48 | HT1-7 |
| ARC 10633 | W112 | Ind | India | 28.6 | 77.2 | HT1-7 |
| Hsin Hsing Pai Ku | W132 | IndI | Taiwan | 25.02 | 121.38 | HT1-7 |
| TD 70 | W133 | Ind | Thailand | 13.8 | 100.5 | HT1-7 |
| Babaomi | W198 | IndI | China | 39.9 | 116.4 | HT1-7 |
| Suiyangnian | W235 | IndI | Unknown | NA | NA | HT1-7 |
| RUSTYLATE | W246 | Ind | Unknown | NA | NA | HT1-7 |
| Yunjiang35 | W266 | IndII | Unknown | NA | NA | HT1-7 |
| IR65600-27-1-2-2 | W269 | Ind | Philippines | 14.6 | 121 | HT1-7 |
|  |  |  |  |  |  |  |
| Dular | C015 | Aus | Heilongjiang | 45.45 | 126.41 | HT11-2 |
| Dular | GP47 | Aus | India | NA | NA | HT11-2 |
| Bena Jhupi | GP530 | Aus | India | NA | NA | HT11-2 |
| ARC 7043 | GP62 | Aus | India | NA | NA | HT11-2 |
| Nang Bang Bentre | W094 | Aus | Vietnam | 21.03 | 105.85 | HT11-2 |
| Jhona349 | W214 | Aus | Pakistan | 33.4 | 73.08 | HT11-2 |
| Karnal Local | W215 | Aus | India | 28.6 | 77.2 | HT11-2 |
| Sada solay | W280 | Aus | Pakistan | 33.4 | 73.08 | HT11-2 |
