# Supplementary material for: Unmasking Novel Loci for Internal Phosphorus Utilization Efficiency in Rice Germplasm through Genome-Wide Association Analysis
Source: PLoS One. 2015 Apr 29;10(4):e0124215. doi: 10.1371/journal.pone.0124215 (PMC4414551; doi:10.1371/journal.pone.0124215)
Supplement: S5 Table — Reference columns show their regulation under P-deficiency in roots (R) and shoots (S), given as fold change in mRNA transcript abundance relative to P-sufficient plants (p>0.05, NS; p<0.05, *; p<0.01 **; p<0.001 ***). Transcripts not detected or reported are represented by ‘na’. Variation that was not determined is represented by ‘ND’. (DOC) [file pone.0124215.s010.doc]

**Table S5.** Candidate genes at the highly significant peak on chromosome 1 (7.23-7.36 Mb) and their regulation under P-deficiency in roots (R) and shoots (S), given as fold change in mRNA transcript abundance relative to P-sufficient plants (p>0.05, NS; p<0.05, *; p<0.01 **; p<0.001 ***). Transcripts not detected or reported are represented by ‘na’. SNPs within 1 kb of promoter region are represented as ‘P-SNPs’ and non-synonymous SNPs as ‘NP-SNPs’

| MSU_LOC: | MSU_5' MSU_3' | MSU_Annotation | Pariasca-Tanaka et al. (2010) | Zheng et al. (2009) | rare specific SNP variation |
| --- | --- | --- | --- | --- | --- |
| LOC_Os01g13000 | 7224223 7215513 | ethylene-responsive protein related, putative, expressed | NS | NS | P-SNPs: 0 NS-SNPs: 0 |
| LOC_Os01g13010 | 7235450 7235012 | hypothetical protein | na | na | P-SNPs: 2 NS-SNPs: 0 |
| LOC_Os01g13020 | 7238721 7239957 | hypothetical protein | na | na | P-SNPs: 6 NS-SNPs: 2 (stop codon) |
| LOC_Os01g13024 | 7245003 7243980 | expressed protein | NS | na | P-SNPs: 4 NS-SNPs: 0 |
| LOC_Os01g13030 | 7248761 7253629 | OsIAA3 - Auxin-responsive Aux/IAA gene family member, expressed | 0.79 ** (S) | 0.74 * (S) | P-SNPs: 4 NS-SNPs: 1 |
| LOC_Os01g13040 | 7258786 7259671 | expressed protein | na | na | P-SNPs: 5 NS-SNPs: 0 |
| LOC_Os01g13050 | 7262022 7259671 | long cell-linked locus protein, putative | na | na | P-SNPs: 5 NS-SNPs: 0 |
| LOC_Os01g13060 | 7264228 7272237 | CK1_CaseinKinase_1.1 - CK1 includes the casein kinase 1 kinases, expressed | 0.53 * ( S) | NS | P-SNPs: 5 NS-SNPs: 1 |
| LOC_Os01g13070 | 7275019 7276972 | expressed protein | na | NS | P-SNPs: 2 NS-SNPs: 0 |
| LOC_Os01g13080 | 7279250 7277720 | 60S acidic ribosomal protein, putative, expressed | 2.16 *** (S) | 1.65 * (R) | P-SNPs: 2 NS-SNPs: 0 |
| LOC_Os01g13090 | 7280734 7284992 | nucleic acid binding protein, putative, expressed | NS | NS | P-SNPs: 8 NS-SNPs: 4 |
| LOC_Os01g13100 | 7286907 7293276 | KH domain containing protein, putative, expressed | NS | 1.52 * (R) | P-SNPs: 8 NS-SNPs: 1 |
| LOC_Os01g13120 | 7296672 7293276 | aquaporin protein, putative, expressed | na | na | P-SNPs: 2 NS-SNPs: 0 |
| LOC_Os01g13130 | 7301368 7302788 | aquaporin protein, putative, expressed | NS | NS | P-SNPs: 2 NS-SNPs: 0 |
| LOC_Os01g13140 | 7308906 7306881 | WD domain, G-beta repeat domain containing protein | na | na | P-SNPs: 5 NS-SNPs: 2 |
| LOC_Os01g13150 | 7324145 7331536 | metallo-beta-lactamase family protein, putative, expressed | na | 1.94 *(R) 2.99 *(S) | P-SNPs: 16 NS-SNPs: 14 |
| LOC_Os01g13160 | 7332691 7338244 | expressed protein | 0.81 ** (R) | NS | P-SNPs: 2 NS-SNPs: 4 |
| LOC_Os01g13170 | 7339717 7344465 | ubiquitin-conjugating enzyme E2, putative, expressed | NS | NS | P-SNPs: 15 NS-SNPs: 6 |
| LOC_Os01g13180 | 7345978 7344590 | expressed protein | na | na | P-SNPs: 0 NS-SNPs: 0 |
| LOC_Os01g13190 | 7347563 7351146 | histidinol dehydrogenase, chloroplast precursor, putative, expressed | 0.64 *** (S) | NS | P-SNPs: 0 NS-SNPs: 0 |
| LOC_Os01g13200 | 7356620 7351175 | abscisic acid insensitive 8, putative, expressed | na | NS | P-SNPs: 0 NS-SNPs: 0 |
