# Supplementary material for: Unmasking Novel Loci for Internal Phosphorus Utilization Efficiency in Rice Germplasm through Genome-Wide Association Analysis
Source: PLoS One. 2015 Apr 29;10(4):e0124215. doi: 10.1371/journal.pone.0124215 (PMC4414551; doi:10.1371/journal.pone.0124215)
Supplement: S6 Table — Reference columns show their regulation under P-deficiency in roots (R) and shoots (S), given as fold change in mRNA transcript abundance relative to P-sufficient plants (p>0.05, NS; p<0.05, *; p<0.01 **; p<0.001 ***). Transcripts not detected or reported are represented by ‘na’. Variation that was not determined is represented by ‘ND’. (DOC) [file pone.0124215.s011.doc]

**Table S6.** Candidate genes at the highly significant peak on chromosome 4 (17.7-17.85 Mb) and their regulation under P-deficiency in roots (R) and shoots (S), given as fold change in mRNA transcript abundance relative to P-sufficient plants (p>0.05, NS; p<0.05, *; p<0.01 **; p<0.001 ***). Transcripts not detected or reported are represented by ‘na’. Variation that was not determined is represented by ‘ND’.

| MSU_LOC: | MSU_5' MSU_3' | MSU_Annotation | Pariasca-Tanaka et al. (2010) | Zheng et al. (2009) | rare specific SNP variation |
| --- | --- | --- | --- | --- | --- |
| LOC_Os04g29950 | 17696813 17693417 | wall-associated receptor kinase, putative | na | 0.87* (S) | ND |
| LOC_Os04g29960 | 17705322 17702355 | OsWAK43 - OsWAK receptor-like protein kinase, expressed | na | NS | ND |
| LOC_Os04g29970 | 17710624 17709772 | hypothetical protein | na | 0.82** (R) | ND |
| LOC_Os04g29980 | 17714077 17712749 | transposon protein, putative, CACTA, En/Spm sub-class | na | na | ND |
| LOC_Os04g29990 | 17719615 17716187 | OsWAK44 - OsWAK receptor-like protein kinase | na | NS | ND |
| LOC_Os04g30000 | 17731965 17732538 | hypothetical protein | na | NS | ND |
| LOC_Os04g30020 | 17740574 17740976 | expressed protein | na | NS | ND |
| LOC_Os04g30050 | 17771906 17772420 | hypothetical protein | na | 0.76* (S) | ND |
| LOC_Os04g30060 | 17773846 17772809 | wall-associated receptor kinase-like 10 precursor, putative | na | 0.86* (R) | ND |
| LOC_Os04g30070 | 17775001 17775342 | hypothetical protein | na | 0.82* (S) | ND |
| LOC_Os04g30080 | 17779323 17779093 | retrotransposon protein, putative, Ty3-gypsy subclass | na | na | ND |
| LOC_Os04g30090 | 17784884 17783567 | retrotransposon protein, putative, unclassified | na | na | ND |
| LOC_Os04g30100 | 17787881 17788654 | transposon protein, putative, CACTA, En/Spm sub-class | na | na | ND |
| LOC_Os04g30110 | 17790725 17789889 | wall-associated receptor kinase 3 precursor, putative, expressed | na | 1.24*** (S) | ND |
| LOC_Os04g30120 | 17793489 17792061 | hypothetical protein | na | na | ND |
| LOC_Os04g30130 | 17805259 17799314 | retrotransposon protein, putative, unclassified | na | na | ND |
| LOC_Os04g30140 | 17808544 17809016 | hypothetical protein | na | 0.94** (S) | ND |
| LOC_Os04g30150 | 17817914 17813691 | retrotransposon protein, putative, unclassified | na | na | ND |
| LOC_Os04g30170 | 17839912 17839297 | hypothetical protein | na | na | ND |
| LOC_Os04g30190 | 17850966 17845221 | transposon protein, putative, unclassified | na | na | ND |
| LOC_Os04g30200 | 17851719 17854071 | OsFBL14 - F-box domain and LRR containing protein, expressed | na | na | ND |
