# Supplementary material for: Unmasking Novel Loci for Internal Phosphorus Utilization Efficiency in Rice Germplasm through Genome-Wide Association Analysis
Source: PLoS One. 2015 Apr 29;10(4):e0124215. doi: 10.1371/journal.pone.0124215 (PMC4414551; doi:10.1371/journal.pone.0124215)
Supplement: S7 Table — Reference columns show their regulation under P-deficiency in roots (R) and shoots (S), given as fold change in mRNA transcript abundance relative to P-sufficient plants (p>0.05, NS; p<0.05, *; p<0.01 **; p<0.001 ***). Transcripts not detected or reported are represented by ‘na’. Variation that was not determined is represented by ‘ND’. (DOC) [file pone.0124215.s012.doc]

**Table S7.** Candidate genes at the highly significant peak on chromosome 11 (16.63-16.75 Mb) and their regulation under P-deficiency in roots (R) and shoots (S), given as fold change in mRNA transcript abundance relative to P-sufficient plants (p>0.05, NS; p<0.05, *; p<0.01 **; p<0.001 ***). Transcripts not detected or reported are represented by ‘na’. SNPs within 1 kb of promoter region are represented as ‘P-SNPs’ and non-synonymous SNPs as ‘NP-SNPs’

| MSU_LOC: | MSU_5' MSU_3' | MSU_Annotation | Pariasca-Tanaka et al. (2010) | Zheng et al. (2009) | haplotype specific SNP variation |
| --- | --- | --- | --- | --- | --- |
| LOC_Os11g29470 | 16634045 16631968 | expressed protein | na | na | P-SNPs: 4 NS-SNPs: 4 |
| LOC_Os11g29460 | 16633978 16634722 | hypothetical protein | na | na | P-SNPs: 0 NS-SNPs: 2 |
| LOC_Os11g29480 | 16639220 16640621 | hypothetical protein | na | 0.94* (R) | P-SNPs: 2 NS-SNPs: 2 |
| LOC_Os11g29490 | 16642596 16649451 | plasma membrane ATPase, putative, expressed | NS | NS | P-SNPs: 2 NS-SNPs: 2 (stop codon) |
| LOC_Os11g29500 | 16650610 16649869 | expressed protein | 0.81** (S) | NS | P-SNPs: 4 NS-SNPs: 0 |
| LOC_Os11g29510 | 16655481 16651725 | cysteine-rich receptor-like protein kinase 35 precursor, putative | NS | NS | P-SNPs: 0 NS-SNPs: 0 |
| LOC_Os11g29520 | 16666068 16660482 | NBS-LRR disease resistance protein, putative, expressed | 0.79** (S) | 1.15* (S) 0.96* (R) | P-SNPs: 0 NS-SNPs: 1 |
| LOC_Os11g29530 | 16669766 16675726 | retrotransposon protein, putative, unclassified | na | na | P-SNPs: 0 NS-SNPs: 1 |
| LOC_Os11g29540 | 16678067 16676829 | retrotransposon protein, putative, unclassified | na | na | P-SNPs: 0 NS-SNPs: 0 |
| LOC_Os11g29550 | 16679020 16680775 | retrotransposon protein, putative, unclassified | na | NS | P-SNPs: 0 NS-SNPs: 2 |
| LOC_Os11g29560 | 16683471 16686398 | retrotransposon protein, putative, unclassified | na | na | P-SNPs: 0 NS-SNPs: 0 |
| LOC_Os11g29570 | 16692533 16686728 | retrotransposon protein, putative, Ty3-gypsy subclass | na | na | P-SNPs: 0 NS-SNPs: 0 |
| LOC_Os11g29580 | 16696844 16696596 | hypothetical protein | na | 0.61* (S) 0.63* (R) | P-SNPs: 0 NS-SNPs: 0 |
| LOC_Os11g29600 | 16703209 16702875 | RING zinc finger ankyrin protein, putative | na | na | P-SNPs: 2 NS-SNPs: 0 |
| LOC_Os11g29610 | 16704786 16709042 | retrotransposon protein, putative, Ty1-copia subclass | na | na | P-SNPs: 0 NS-SNPs: 0 |
| LOC_Os11g29620 | 16713786 16713202 | hypothetical protein | na | NS | P-SNPs: 0 NS-SNPs: 0 |
| LOC_Os11g29630 | 16716347 16714381 | expressed protein | na | na | P-SNPs: 1 NS-SNPs: 1 |
| LOC_Os11g29640 | 16719228 16718234 | transposon protein, putative, CACTA, En/Spm sub-class | na | na | P-SNPs: 0 NS-SNPs: 0 |
| LOC_Os11g29650 | 16721934 16719344 | transposon protein, putative, CACTA, En/Spm sub-class | na | na | P-SNPs: 0 NS-SNPs: 0 |
| LOC_Os11g29660 | 16727867 16724563 | transposon protein, putative, CACTA, En/Spm sub-class | na | na | P-SNPs: 0 NS-SNPs: 0 |

**Table S7.** continued; candidate genes around the high PUE peak on chomosome11

| MSU_LOC: | MSU_5' MSU_3' | MSU_Annotation | Pariasca-Tanaka et al. (2010) | Zheng et al. (2009) | haplotype specific SNP variation |
| --- | --- | --- | --- | --- | --- |
| LOC_Os11g29670 | 16734959 16734061 | expressed protein | na | na | P-SNPs: 2 NS-SNPs: 0 |
| LOC_Os11g29680 | 16736941 16739633 | expressed protein | na | na | P-SNPs: 1 NS-SNPs: 1 |
| LOC_Os11g29690 | 16742015 16744015 | oxidoreductase, 2OG-Fe oxygenase family protein, putative, expressed | NS | NS | P-SNPs: 0 NS-SNPs: 0 |
| LOC_Os11g29700 | 16746677 16744495 | expressed protein | 0.56*** (S) | 0.63* (S) | P-SNPs: 2 NS-SNPs: 1 |
