# Supplementary material for: Unmasking Novel Loci for Internal Phosphorus Utilization Efficiency in Rice Germplasm through Genome-Wide Association Analysis
Source: PLoS One. 2015 Apr 29;10(4):e0124215. doi: 10.1371/journal.pone.0124215 (PMC4414551; doi:10.1371/journal.pone.0124215)
Supplement: S8 Table — Reference columns show their regulation under P-deficiency in roots (R) and shoots (S), given as fold change in mRNA transcript abundance relative to P-sufficient plants (p>0.05, NS; p<0.05, *; p<0.01 **; p<0.001 ***). Transcripts not detected or reported are represented by ‘na’. Variation that was not determined is represented by ‘ND’. (DOC) [file pone.0124215.s013.doc]

**Table S8.** Candidate genes at the highly significant peak on chromosome 12 (17.7-17.85 Mb). Reference columns show their regulation under P-deficiency in roots (R) and shoots (S), given as fold change in mRNA transcript abundance relative to P-sufficient plants (p>0.05, NS; p<0.05, *; p<0.01 **; p<0.001 ***). Transcripts not detected or reported are represented by ‘na’. Variation that was not determined is represented by ‘ND’.

| MSU_LOC: | MSU_5' MSU_3' | MSU_Annotation | Pariasca-Tanaka et al. (2010) | Zheng et al. (2009) | haplotype specific SNP variation |
| --- | --- | --- | --- | --- | --- |
| LOC_Os12g42550 | 26396477 26391714 | methyl-CpG binding domain containing protein, putative, expressed | 0.87* (S) | 1.09* (R) | ND |
| LOC_Os12g42570 | 26404411 26399720 | expressed protein | 1.55*** (S) | NS | ND |
| LOC_Os12g42600 | 26421349 26427732 | ubiquitin carboxyl-terminal hydrolase domain containing protein, expressed | na | NS | ND |
| LOC_Os12g42610 | 26444027 26453782 | YABBY domain containing protein, putative, expressed | NS | NS | ND |
| LOC_Os12g42620 | 26457028 26456780 | hypothetical protein | na | 0.72* (S) | ND |
| LOC_Os12g42630 | 26465743 26464577 | transposon protein, putative, Mutator sub-class | 1.35** (S) | NS | ND |
| LOC_Os12g42640 | 26470961 26467261 | retrotransposon protein, putative, Ty3-gypsy subclass | na | NS | ND |
| LOC_Os12g42650 | 26473582 26472675 | pollen preferential protein, putative, expressed | na | NS | ND |
| LOC_Os12g42660 | 26473835 26479946 | AGC_AGC_other_GWLd.1 - ACG kinases include homologs to PKA, PKG and PKC, expressed | NS | NS | ND |
| LOC_Os12g42670 | 26481035 26480146 | expressed protein | NS | NS | ND |
| LOC_Os12g42680 | 26484222 26490180 | retrotransposon protein, putative, unclassified | na | na | ND |
| LOC_Os12g42690 | 26494053 26490502 | retrotransposon protein, putative, unclassified | na | na | ND |
| LOC_Os12g42700 | 26496675 26502438 | expressed protein | 1.45*** (S) | 1.63* (R) | ND |
| LOC_Os12g42710 | 26503078 26505525 | retrotransposon protein, putative, Ty3-gypsy subclass | na | na | ND |

**Table S8.** continued; candidate genes around the high PUE peak on chomosome12

| MSU_LOC: | MSU_5' MSU_3' | MSU_Annotation | Pariasca-Tanaka et al. (2010) | Zheng et al. (2009) | haplotype specific SNP variation |
| --- | --- | --- | --- | --- | --- |
| LOC_Os12g42730 | 26514778 26524013 | mov34/MPN/PAD-1 family protein, expressed | NS | 1.43* (R) | ND |
| LOC_Os12g42720 | 26515155 26509863 | retrotransposon protein, putative, unclassified | 1.25* (S) | na | ND |
| LOC_Os12g42739 | 26528427 26527337 | expressed protein | 1.16* (S) | na | ND |
| LOC_Os12g42750 | 26531016 26529396 | transposon protein, putative, unclassified | na | na | ND |
| LOC_Os12g42760 | 26544351 26547435 | type IIB DNA topoisomerase, putative, expressed | 5.46***(S) 0.79* (R) | 1.49* (R) | ND |
| LOC_Os12g42770 | 26551574 26550292 | hypothetical protein | na | 0.81* (S) | ND |
| LOC_Os12g42780 | 26553621 26553929 | hypothetical protein | na | na | ND |
| LOC_Os12g42790 | 26558486 26559883 | retrotransposon protein, putative, unclassified | na | na | ND |
| LOC_Os12g42800 | 26560923 26561606 | retrotransposon protein, putative, unclassified | na | na | ND |
| LOC_Os12g42810 | 26566126 26571177 | mov34/MPN/PAD-1 family protein, expressed | 0.76* (S) | NS | ND |
| LOC_Os12g42820 | 26575887 26577989 | SWITCH1 splice variant S, putative | na | 0.85* (S) | ND |
| LOC_Os12g42830 | 26580135 26583625 | conserved hypothetical protein | na | NS | ND |
| LOC_Os12g42840 | 26584635 26588175 | expressed protein | na | 1.18* (S) | ND |
| LOC_Os12g42850 | 26592524 26587454 | amino acid permease, putative, expressed | 0.84** (S) 0.96* (R) | 0.79* (R) | ND |
| LOC_Os12g42860 | 26599020 26596310 | 2-aminoethanethiol dioxygenase, putative, expressed | 0.88* (S) 0.84* (R) | 1.32* (S) 0.9* (R) | ND |
| LOC_Os12g42870 | 26606944 26602092 | Mur ligase family protein, putative, expressed | NS | NS | ND |
| LOC_Os12g42876 | 26613088 26617173 | 5-methyltetrahydropteroyltriglutamate--homocysteine methyltransferase, putative, expressed | NS | na | ND |
| LOC_Os12g42884 | 26623587 26629389 | 5-methyltetrahydropteroyltriglutamate--homocysteine methyltransferase, putative, expressed | 0.88* (S) 0.84* (R) | 1.11* (S) 1.33* (R) | ND |
| LOC_Os12g42900 | 26631216 26629559 | lysine-rich arabinogalactan protein 19 precursor, putative, expressed | 1.24* (R) | 0.87* (R) | ND |
| LOC_Os12g42910 | 26635551 26637596 | sodium/calcium exchanger protein, putative, expressed | na | na | ND |
| LOC_Os12g42920 | 26638839 26640139 | expressed protein | na | NS | ND |
| LOC_Os12g42930 | 26644749 26641060 | hypothetical protein | na | 1.06* (R) | ND |
| LOC_Os12g42940 | 26647548 26649730 | transposon protein, putative, unclassified | na | na | ND |
| LOC_Os12g42950 | 26651130 26652215 | hypothetical protein | na | NS | ND |

**Table S8.** Continued; candidate genes around the high PUE peak on chomosome12

| MSU_LOC: | MSU_5' MSU_3' | MSU_Annotation | Pariasca-Tanaka et al. (2010) | Zheng et al. (2009) | haplotype specific SNP variation |
| --- | --- | --- | --- | --- | --- |
| LOC_Os12g42960 | 26655802 26653186 | expressed protein | NS | NS | ND |
| LOC_Os12g42970 | 26659915 26662912 | GATA zinc finger domain containing protein, expressed | NS | 1.86* (R) | ND |
| LOC_Os12g42980 | 26669749 26665045 | cysteine synthase, putative, expressed | 0.77* (S) | 1.06* (R) | ND |
| LOC_Os12g42990 | 26675783 26674244 | retrotransposon protein, putative, unclassified | na | 0.81* (R) | ND |
| LOC_Os12g43000 | 26684576 26680941 | retrotransposon protein, putative, unclassified, expressed | 0.69* (S) | NS | ND |
| LOC_Os12g43020 | 26688201 26693976 | retrotransposon protein, putative, unclassified | na | na | ND |
| LOC_Os12g43034 | 26698037 26695297 | retrotransposon protein, putative, unclassified | na | na | ND |
| LOC_Os12g43050 | 26708907 26702127 | retrotransposon protein, putative, Ty3-gypsy subclass | na | na | ND |
| LOC_Os12g43060 | 26710780 26716787 | retrotransposon protein, putative, unclassified | na | NS | ND |
| LOC_Os12g43070 | 26718582 26718956 | protein transport protein Sec61, putative | na | NS | ND |
| LOC_Os12g43080 | 26719386 26722063 | conserved hypothetical protein | na | NS | ND |
| LOC_Os12g43090 | 26723888 26725585 | expressed protein | NS | 0.84* (R) | ND |
| LOC_Os12g43100 | 26728768 26725980 | deoxyhypusine hydroxylase, putative, expressed | na | 0.81* (S) 1.5* (R) | ND |
| LOC_Os12g43110 | 26734618 26735291 | OsSAUR58 - Auxin-responsive SAUR gene family member, expressed | NS | 1.8* (S) 2.6** (R) | ND |
| LOC_Os12g43120 | 26745515 26737124 | expressed protein | na | NS | ND |
| LOC_Os12g43130 | 26749996 26746885 | phytoene synthase, chloroplast precursor, putative, expressed | 3.79*** (R) | 0.2*** (S) 2.01** (R) | ND |
| LOC_Os12g43140 | 26759679 26760353 | late embryogenesis abundant protein D-34, putative, expressed | na | 0.46* (R) | ND |
| LOC_Os12g43150 | 26764744 26760625 | hypothetical protein | na | NS | ND |
| LOC_Os12g43160 | 26767810 26769148 | retrotransposon protein, putative, Ty3-gypsy subclass | na | na | ND |
| LOC_Os12g43165 | 26771227 26775573 | retrotransposon protein, putative, Ty3-gypsy subclass, expressed | na | na | ND |
| LOC_Os12g43179 | 26784854 26778901 | retrotransposon protein, putative, Ty3-gypsy subclass | na | na | ND |
| LOC_Os12g43200 | 26787221 26787580 | conserved hypothetical protein | na | na | ND |
| LOC_Os12g43220 | 26795471 26788128 | retrotransposon protein, putative, unclassified | na | na | ND |
| LOC_Os12g43230 | 26802366 26797053 | retrotransposon protein, putative, Ty3-gypsy subclass | na | na | ND |

**Table S8.** Continued; candidate genes around the high PUE peak on chomosome12

| MSU_LOC: | MSU_5' MSU_3' | MSU_Annotation | Pariasca-Tanaka et al. (2010) | Zheng et al. (2009) | haplotype specific SNP variation |
| --- | --- | --- | --- | --- | --- |
| LOC_Os12g43240 | 26806974 26802638 | retrotransposon protein, putative, Ty3-gypsy subclass | na | na | ND |
| LOC_Os12g43250 | 26814767 26812941 | retrotransposon protein, putative, unclassified | na | na | ND |
| LOC_Os12g43260 | 26819936 26817317 | retrotransposon protein, putative, unclassified | na | na | ND |
| LOC_Os12g43270 | 26824462 26831682 | retrotransposon protein, putative, Ty3-gypsy subclass | na | na | ND |
| LOC_Os12g43280 | 26835281 26832354 | retrotransposon protein, putative, unclassified | na | na | ND |
| LOC_Os12g43290 | 26838552 26836241 | hypothetical protein | na | 1.13* (S) | ND |
| LOC_Os12g43300 | 26839149 26844602 | expressed protein | na | 0.85* (R) | ND |
| LOC_Os12g43310 | 26846388 26846705 | hypothetical protein | na | NS | ND |
| LOC_Os12g43320 | 26848776 26849141 | hypothetical protein | na | NS | ND |
| LOC_Os12g43330 | 26853476 26853817 | hypothetical protein | na | NS | ND |
| LOC_Os12g43340 | 26856653 26855162 | actin-depolymerizing factor, putative, expressed | na | 0.31* (S) 0.38* (R) | ND |
| LOC_Os12g43350 | 26861456 26862809 | expressed protein | 1.56* (R) | 1.59* (S) | ND |
| LOC_Os12g43400 | 26890545 26891096 | hypothetical protein | na | NS | ND |
